# Comprehensive and comparative lipidome analysis of *Vitis vinifera* L. cv. Pinot Noir and Japanese indigenous *V*. *vinifera* L. cv. Koshu grape berries

**DOI:** 10.1371/journal.pone.0186952

**Published:** 2017-10-20

**Authors:** Kayo Arita, Taro Honma, Shunji Suzuki

**Affiliations:** 1 Laboratory of Fruit Genetic Engineering, The Institute of Enology and Viticulture, University of Yamanashi, Kofu, Yamanashi, Japan; 2 Laboratory of Toxicology, Faculty of Pharma-Science, Teikyo University, Kaga, Itabashi, Tokyo, Japan; University of Illinois, UNITED STATES

## Abstract

*Vitis vinifera* cv. Koshu is an indigenous grape cultivar that has been cultivated for more than a thousand years in Japan and one of the most important cultivars in white winemaking. To improve Koshu wine quality, it is necessary to identify the metabolites in Koshu berry. We conducted a comprehensive and comparative lipidome analysis of Koshu and Pinot Noir berries cultivated in the same location in Japan using GC-MS/MS for fatty acids and LC-MS for glycerolipids and glycerophospholipids. Koshu skins and juices contained 22 and 19 fatty acids, respectively, whereas 23 and 20 fatty acids were detected in Pinot Noir skins and juices. C22:6n3 and C24:0 contents in Koshu skins were two and three times higher than those in Pinot Noir skins. C24:0 content in Koshu juices was also higher than that in Pinot Noir juices. Forty-nine lipid components (six digalactosyldiacylglycerols, one monogalactosyldiacylglycerol, 10 phosphatidylcholines, 12 phosphatidylethanolamines, and 20 triglycerides) were detected in Pinot Noir and Koshu skins. Strong peaks were observed for MGDG 36:6, DGDG 36:6, PC 34:2, PC 36:5, TG 54:6, TG 54:7, and TG 54:8 in Koshu skins. The contents of 36 of the 49 lipid components were significantly higher in Pinot Noir skins than Koshu skins. Pinot Noir skins contained more lipids whose alkyl chains have more than 18 carbons than Koshu skins. Further analysis of both lipid profiles revealed that the number of double bonds in a fatty acid molecule in Pinot Noir skins and juices was significantly larger than that in Koshu skins and juices. A strong relationship exists between the heat requirement of grapevine cultivars and the level of fatty acid desaturation. C18-fatty acids were the major components in Koshu and Pinot Noir berries. The expression levels of C18-fatty acid desaturases regulated the accumulation of C18-unsaturated fatty acids in berry skins. The loss of C18:3 in Koshu berries at the end of ripening was observed. Koshu might effectively convert C18:3 into (*Z*)-hex-3-enal for the production of C6-aroma compounds. These findings by the lipidome analysis are expected to contribute to the improvement of Koshu wine aroma and breeding strategies of cold-tolerant Koshu grapevines.

## Introduction

*Vitis vinifera* L. cv. Koshu is an indigenous grape cultivar that has been cultivated for more than a thousand years in Central Japan. Koshu is a hybrid that is 70% *V*. *vinifera* and 30% Chinese wild species *V*. *davidii* or a closely related species [1]. The characteristics of Koshu berries differ from those of European *V*. *vinifera* berries. For example, berry size of Koshu is two times larger than those of Chardonnay and Pinot Noir. Genetic analysis demonstrated that Koshu has the red and white alleles of *MybA1* gene, indicating that Koshu is genetically a member of black/red cultivars [2]. However, the short insertion in the second intron of the *MybA1* gene in the red allele decreases *MybA1* gene transcription activity, generating purple skin color in Koshu berry. Based on the genetic background, Koshu white wines have higher total phenolic content than white wines made from European *V*. *vinifera* white cultivars and show slight astringency [3].

To characterize Koshu berry metabolites, we determined the transcriptional profiles during berry development [4], the amino acid profiles during berry development [4], and the phenolic compound profiles at harvest [5]. Those studies proved that Koshu berry metabolites are different from the metabolites of European *V*. *vinifera* cultivars. Since Koshu is one of the most important cultivars in white winemaking, we focused on, in the present study, the lipid profiles of Koshu berry skin and juice, but not seed, at the end of the ripening period and performed a lipidomic comparison of Koshu and Pinot Noir berries to characterize Koshu berry metabolites. Pinot Noir was used as the control cultivar because the successful whole genome sequencing of Pinot Noir [6] has accelerated grapevine metabolic research in broad research areas.

During fermentation, wine yeasts utilize free fatty acids but not complex lipids that bind one or multiple fatty acids to a backbone molecule [7]. The levels of free fatty acids in grape juice affect the production of volatile compounds by wine yeasts [8]. *V*. *vinifera* cultivars have high levels of unsaturated fatty acids compared with other *Vitis* varieties [9]. Linoleic acid (C18:2) and linolenic acid (C18:3) are the major components in *V*. *vinifera* berry irrespective of skin color [9]. C6 and C9 aldehydes, which are important volatiles responsible for the ‘green aroma’ in Cabernet Sauvignon berry and wine, are generated from linoleic acid and linolenic acid by oxidative cleavage [10]. However, excess linoleic acid content in juice reduces 3-mercaptohexyl acetate level in Sauvignon Blanc wine [11]. 3-Mercaptohexyl acetate and 3-mercaptohexan-1-ol are important sources of passion fruit aroma in Koshu wine [12]. Although the extraction of aromatic compounds by skin contact has contributed to the establishment of the original style of Koshu wine [13], the qualitative and quantitative analyses of lipids in Koshu berry at the end of the ripening period should be accomplished to improve current understanding of Koshu wine aromas, especially the expression of aroma compounds originating from Koshu berry.

LIPID MAPS (http://www.lipidmaps.org/) classifies lipids into eight categories: fatty acyls, glycerolipids, glycerophospholipids, sphingolipids, sterol lipids, prenol lipids, saccharolipids, and polyketides. Their structural complexity has complicated comprehensive analysis in grape berry. Although recent technological advances have enabled the comprehensive profiling of lipidome in whole berry [14] and juice [15], knowledge of lipid composition in juice and skin of grape berry is still limited. In the present study, using GC-MS/MS for free fatty acids and LC-MS for glycerolipids and glycerophospholipids, comprehensive lipid profiling of Koshu and Pinot Noir berry skin and juice at the end of the ripening period was conducted and a comparative analysis was performed for Koshu and Pinot Noir berries to understand Koshu lipid metabolism.

## Materials and methods

### Chemicals

Caprylic acid (C8:0), capric acid (C10:0), lauric acid (C12:0), myristic acid (C14:0), stearic acid (C18:0), arachidonic acid (C20:4n6), and methyl-d3 stearate standard were obtained from Wako Pure Chemicals (Tokyo, Japan).

### Grape juice and skin samples

*Vitis vinifera* cvs. Koshu and Pinot Noir were cultivated in the experimental vineyard of The Institute of Enology and Viticulture, University of Yamanashi, Japan. The grapevines were approximately 30 years old and trained to the Guyot-style system. Five grapevines were used for preparing skin and juice samples.

As an index of maturity of grape berries, Brix of juice sampled from grape berries was measured with a refractometer (Atago, Tokyo, Japan). Five bunches were collected from each grapevine at harvest (end of ripening period; August 10, 2013 for Pinot Noir and October 2, 2013 for Koshu). Fruit juices were obtained by hand-pressing the five bunches. After centrifugation at 16,000 × g, the supernatant was filtered through 0.2 μm membrane filter (Pall, East Hills, NY) and the filtrate was subjected to analysis.

Fifty fresh berries were randomly sampled from each grapevine at the end of the ripening period and skins were peeled off by using tweezers. The skins were dried by lyophilization for 24 h and then pulverized in liquid nitrogen.

### Sample preparation for fatty acid recovery test from samples supplemented with fatty acids

To determine the recovery rates of fatty acids, a recovery test of juice supplemented with fatty acids was performed. Koshu juice (1.5 mL) was supplemented with 15 μL of fatty acid solution (caprylic acid, capric acid, lauric acid, myristic acid, stearic acid, and arachidonic acid, each 50 μg/mL). Blank samples were not supplemented with fatty acids. Four mL of methanol and 2 mL of 0.1% 2,6-di-tert-butyl-p-cresol were added to the juice. The juice was mixed well and then 4 mL of 0.1% 2,6-di-tert-butyl-p-cresol and 4 mL of 1% formic acid were added. After centrifugation at 1,500 × g for 5 min, the organic layer was collected.

For skin samples, 0.05 g of pulverized Koshu skin sample was supplemented with 15 μL of the fatty acid solution and 300 μL of Milli-Q water. To the sample were added 800 μL of methanol and 400 μL of 0.1% 2,6-di-tert-butyl-p-cresol. The sample was mixed well and then 800 μL of 0.1% 2,6-di-tert-butyl-p-cresol and 800 μL of 1% formic acid were added. After vortexing for 30 s and centrifugation at 1,500 × g for 5 min, the organic layer was collected.

Solid-phase extraction of the organic layers was performed using a Supelclean ENVI-Carb SPE tube (wt 250 mg/vol. 6 mL, Supelco, Bellefonte, PA). Washing was done with 5 mL of chloroform. The eluate was obtained with 10 mL of chloroform and then dried by nitrogen purging. The dry solid was dissolved in 90 μL of 20% methanol/benzene, and this was followed by the addition of 45 μL of trimethylsilyldiazomethane. Finally, 15 μL of methyl-d3 stearate standard (100 μg/mL) was added to the solution. The solution was subjected to GC-MS/MS as described below. Three samples of Koshu juice or skin were used for the recovery test.

### Sample preparation for quantification of fatty acids in juices and skins

Koshu and Pinot Noir juices and skins were used. 1.5 mL of juice was supplemented with 15 μL of methyl-d3 stearate standard (100 μg/mL). Four mL of methanol and 2 mL of 0.1% 2,6-di-tert-butyl-p-cresol were added to the juice. The juice was mixed well and then 4 mL of 0.1% 2,6-di-tert-butyl-p-cresol and 4 mL of 1% formic acid were added. After centrifugation at 1,500 × g for 5 min, the organic layer was collected.

For skin samples, 0.05 g of pulverized skin sample was supplemented with 15 μL of methyl-d3 stearate standard (100 μg/mL) and 300 μL of Milli-Q water. 800 μL of methanol and 400 μL of 0.1% 2,6-di-tert-butyl-p-cresol were added to the sample. The sample was mixed well and then 800 μL of 0.1% 2,6-di-tert-butyl-p-cresol and 800 μL of 1% formic acid were added. After vortexing for 30 s and centrifugation at 1,500 × g for 5 min, organic layer was collected.

Solid-phase extraction of the organic layers was performed using Supelclean ENVI-Carb SPE tube (wt 250 mg/vol. 6 mL). Washing was done with 5 mL of chloroform. The eluate was obtained with 10 mL of chloroform and then dried by nitrogen purging. The dry solid was dissolved in 90 μL of 20% methanol/benzene and this was followed by the addition of 45 μL of trimethylsilyldiazomethane. The solution was subjected to GC-MS/MS as described below. Five samples of each juice or skin were used for quantification of free fatty acids in juices or skins of Koshu and Pinot Noir berries.

### GC-MS/MS

The quantitative analysis of fatty acids was performed using GC-2010 Plus with the GC-MS TQ8030 system (Shimadzu, Kyoto, Japan). GC-MS/MS conditions were as follows: Omegawax 250 column (30 m × 250 μm × 0.25 μm, Supelco); helium carrier gas; linear velocity, 39 cm/s; oven temperature, 50°C (3 min followed by 20°C up/min), 170°C (0 min followed by 2°C up/min), 240°C (5 min); ion source temperature, 200°C; interface temperature, 250°C. The conditions for multiple reaction monitoring (MRM) are listed in S1 Table. Because the separation of C18:1n9c and C18:1n9t peaks by our method was difficult, both contents were summed as C18:1n9c/C18:1n9t.

### Recovery rate of supplemented fatty acids in fatty acid recovery test

For the fatty acid recovery test, caprylic acid, capric acid, lauric acid, myristic acid, stearic acid, and arachidonic acid were quantified in juice and skin samples supplemented with these fatty acids. The peaks for fatty acids were identified as shown in S2 Table, and peaks with signal-to-noise (S/N) ratios higher than 3 were obtained. Peak areas were determined and then each fatty acid was quantified using the internal standard method with methyl-d3 stearate. The recovery rate of each fatty acid was calculated by subtracting the average value of fatty acid in blank samples from each value of fatty acid in samples supplemented with fatty acids.

### Quantification of fatty acids in juices and skins

Fatty acid methyl esters in samples were identified by comparing their retention times with those of authentic standards (F.A.M.E. Mix C4-C24, Supelco). The list of standards is shown in S3 Table. Peak areas obtained by GC-MS/MS were determined and each fatty acid was quantified using the internal standard method with methyl-d3 stearate. The limit of quantification and the concentration range of each fatty acid in juice and skin samples were calculated from the calibration curve created using F.A.M.E. Mix C4-C24 and are listed in S4 and S5 Tables, respectively. Fatty acid contents were calculated using the following formula:

$$Fatty\;acid\;in\;juice\;(ng/mL)=\frac{concentration\;of\;fatty\;acid\;in\;juice\;(ng/mL)\;\times \;0.15\;(mL)}{1.5\;(mL)}$$

$$Fatty\;acid\;in\;skin\;(\mu g/g)=\frac{concentration\;of\;fatty\;acid\;in\;skin\;(\mu g/mL)\;\times \;0.15\;(mL)}{2a}$$

### Sample preparation for preliminary analysis of lipid components in skins

Pulverized Koshu skin samples (5, 10, 15, and 20 mg) were mixed with 250 μL of methanol/dichloromethane (1:1) and the mixture was shaken at 2,500 rpm for 5 min. A 250 μL portion of methanol was added and the mixture was again shaken at 2,500 rpm for 5 min. After centrifugation at 10,000 × g for 5 min, the mixture was passed through 0.2-μm PTFE membrane filter (Millipore, Milford, MA). To 200 μL of the filtrate was added 200 μL of methanol/dichloromethane (2:1) and the solution was subjected to LC-MS measurement as described below. Three samples were used for the preliminary analysis.

### Sample preparation for quantification of lipid components in skins

Pulverized Koshu and Pinot Noir skin samples (20 mg) were added to 250 μL of methanol/dichloromethane (1:1) and the mixture was shaken at 2,500 rpm for 5 min. A 250 μL portion of methanol was added and the mixture was again shaken at 2,500 rpm for 5 min. After centrifugation at 10,000 × g for 5 min, the mixture was passed through 0.2-μm PTFE membrane filter (Millipore). To 200 μL of the filtrate was added 200 μL of methanol/dichloromethane (2:1) and the solution was subjected to LC-MS measurement as described below. Five samples of Koshu or Pinot Noir skin samples were used. The relative contents of each lipid component were represented as peak areas.

### LC-MS

Qualitative and quantitative analyses of lipid components were performed using a LC-MS system (LC:Prominence, Shimadzu; MS:LTQ Orbitrap XL, Thermo Fisher Scientific, Waltham, MA). LC and mass spectrometry conditions were as follows:

LC conditions: L-column2 ODS column (150 mm × 2.1 mm, 3 μm particle diameter, CERI, Tokyo, Japan); column oven temperature, 40°C; mobile phase A (distilled water containing 0.1% (v/v) formic acid); mobile phase B (acetone); flow rate, 0.2 mL/min. Linear gradient program was set from 50% of phase A to 2% of phase A during a 10-min period followed by a program from 2% of phase A to 50% of phase A during a 28.1-min period; injection volume, 3 μL.

MS conditions: All mass spectrometric data were obtained in the electrospray ionization positive-detection mode in a data-dependent fashion. Survey scans were acquired from *m*/*z* 300–1,500 and the resolution for full scan was 30,000.

### Data analysis of lipid components in skins

Data analysis was performed using MZmine 2 software [16]. The parameters for MZmine 2 were as follows: minimum peak height, over 10,000; peak width, 0.2–3.5 min; mass accuracy, 5 ppm; peak deconvolution, local minimum search; join alignment; allowable error of retention time, 0.2 min; allowable error of *m*/*z*, 5 ppm. Lipid species were predicted from the MS/MS spectrum for each peak. To compare the contents of each lipid component between Koshu and Pinot Noir skins, the mean and the standard deviation of the peak area of each lipid component for five skin samples were calculated.

### RNA isolation from berry skins

Fifteen fresh berries of Koshu and Pinot Noir were randomly collected from each grapevine at two weeks post véraison and at harvest. Skins were peeled off from the berries by using tweezers. The skins were placed in a mortar containing liquid nitrogen and homogenized with a pestle. Total RNA isolation from the pulverized skins was performed with a Fruit-mate for RNA Purification (Takara, Otsu, Japan), followed by a NucleoSpin RNA Plant (Takara) for RNA purification according to the manufacturer’s instructions.

### Real-time RT-PCR analysis

cDNA synthesis from total RNA was performed with a PrimeScript RT Reagent Kit with gDNA Eraser (Takara). Real-time RT-PCR was done using an SYBR Premix Ex Taq II (Takara). The conditions for real-time RT-PCR were as follows: 37°C for 15 min and 85°C for 5 s for reverse transcription, and then 40 cycles at 95°C for 5 s and at 60°C for 45 s for PCR amplification. Nucleotide sequences of the primers used in this study were as follows: *V*. *vinifera* stearoyl-[acyl-carrier-protein] 9-desaturase primers (5’-CTTGATGGGGTGAGAGATGAGA-3’ and 5’-ACCCAACCAGAAAGATAGAGATAGG-3’, *V*. *vinifera* stearoyl-[acyl-carrier-protein] 9-desaturase 6, chloroplastic (LOC100255873), mRNA, GenBank accession no. XM_002264759), *V*. *vinifera* omega-3 fatty acid desaturase primers (5’-GGTATCGTGGAAAGGAATGGAG-3’ and 5’-GTGATAGTGCGGGATTTGAGG-3’, *V*. *vinifera* omega-3 fatty acid desaturase, chloroplastic (LOC100247097), mRNA, GenBank accession no. XM_002264314), *V*. *vinifera* omega-6 fatty acid desaturase primers (5’-CAATTCGGCCTTGGATGTCT-3’ and 5’-TGCCAAACTTATCTTCACCCTCTT-3’, *V*. *vinifera* omega-6 fatty acid desaturase, chloroplastic (LOC100248377), mRNA, GenBank accession no. XM_003634815), *V*. *vinifera* lipoxygenase primers (5’-GCAAATCAAAGGGACAACGCTGTATGG-3’ and 5’-TGCTTCCACTGCGGCTTCC-3’, *V*. *vinifera* lipoxygenase (LOXA) mRNA, GenBank accession no. FJ858255), *V*. *vinifera* fatty acid hydroperoxide lyase primers (5’-AAGTACACCGGCGACATTCGAG-3’ and 5’-AGCTCTTTACCCTGGCGTGTTG-3’, *V*. *vinifera* fatty acid hydroperoxide lyase 1 mRNA, GenBank accession no. HM627632) and *V*. *vinifera* actin primers (5’-CAAGAGCTGGAAACTGCAAAGA-3’ and 5’-AATGAGAGATGGCTGGAAGAGG-3’, *V*. *vinifera* actin 1 mRNA, GenBank accession no. AF369524). Dissociation curves were analyzed to verify the specificity of the amplification reaction using Thermal Cycler Dice Real Time System Single Software ver. 3.00 (Takara). The expression level of each gene was determined as the number of amplification cycles needed to reach a fixed threshold using the standard curve method.

### Statistical analysis

For lipidome analysis, data are presented as means ± standard deviation of five biological replicates. Five fruit juice samples were prepared by hand-pressing five bunches, respectively. Five skin samples were obtained from 15 fresh berries randomly sampled from each grapevine, respectively. For gene expression analysis, data are presented as means ± standard deviation of five biological replicates. Five skin samples were obtained from 15 fresh berries randomly sampled from each grapevine, respectively. Statistical analysis was performed with the Student’s *t*-test using Excel Statistics software 2012 (Social Survey Research Information, Tokyo, Japan)

## Results

### Fatty acid recovery test for Koshu and Pinot Noir samples supplemented with fatty acids

The varietal difference in lipidome analysis could be generated by a difference in the ripening progression of the two varieties. First, we provided the ripening progression by Brix to facilitate comparative lipidome analysis of Pinot Noir and Koshu. Brix of Pinot Noir berries were 15.6 ± 0.4 at July 26, 17.9 ± 0.4 at August 10 and 18.0 ± 1.8 at August 25, while Brix of Koshu berries were 14. 6 ± 0.9 at September 14, 16.0 ± 1.4 at October 2 and 16.8 ± 1.1 at October 16. Based on Brix, we judged that each end of ripening period was August 10 for Pinot Noir and October 2 for Koshu, respectively, and applied the berries at August 10 for Pinot Noir and October 2 for Koshu to lipidome analysis.

Representative chromatograms are shown in S1 Fig. Peak areas were measured and the recovered amounts of supplemented fatty acids (C8:0, C10:0, C12:0, C14:0, C18:0, and C20:4n6) were calculated from the calibration curve created from the standard. Recovered amounts, recovery rates, and coefficients of variation (CVs) for juice and skin samples supplemented with fatty acids are summarized in S6 Table. The recovery rates ranged from 83% to 115% irrespective of the sample and the supplemented fatty acid. As the CVs were less than 8 in the recovery test, the reproducibility of the fatty acid recovery from juice and skin samples was excellent. The results suggested that the sample preparation for the quantification of free fatty acids in juices and skins was appropriate.

### Fatty acids in Koshu and Pinot Noir skins and juices

**Fig 1:**
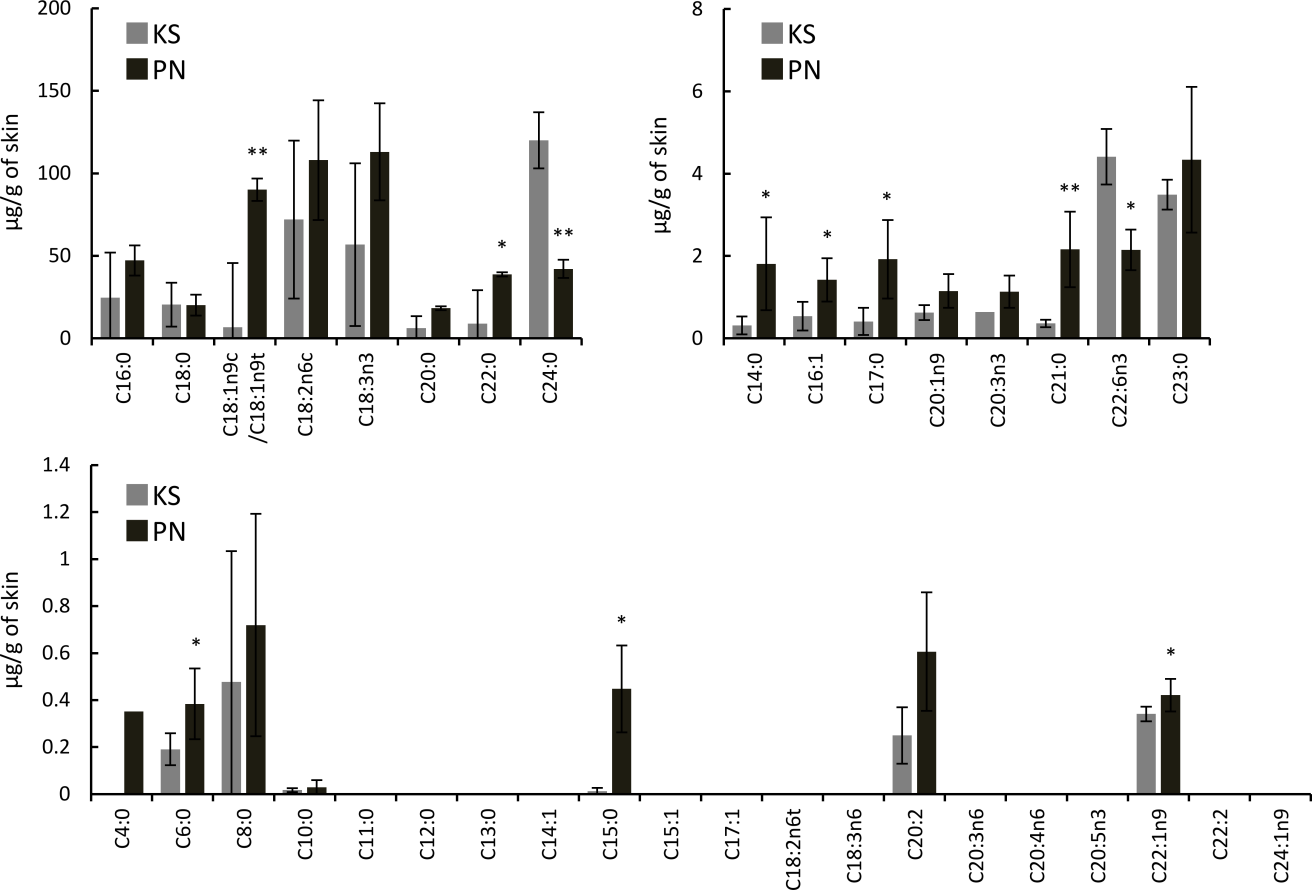
Fatty acids in Koshu and Pinot Noir skins. Data are shown as means ± standard deviation for five independent samples. KS, Koshu. PN, Pinot Noir. *p < 0.05 between Koshu and Pinot Noir skins. **p < 0.01 between Koshu and Pinot Noir skins.

Koshu skins contained 22 fatty acids, whereas Pinot Noir skins contained 23 fatty acids (Fig 1). C4:0 was detected in only Pinot Noir skins, and its content was very low. The contents of C6:0, C14:0, C15:0, C16:1, C17:0, C18:1n9c/C18:1n9t, C21:0, C22:0, and C22:1n9 in Pinot Noir skins were significantly higher than those in Koshu skins. C22:6n3 and C24:0 contents in Koshu skins were two and three times higher than those in Pinot Noir skins, respectively.

**Fig 2:**
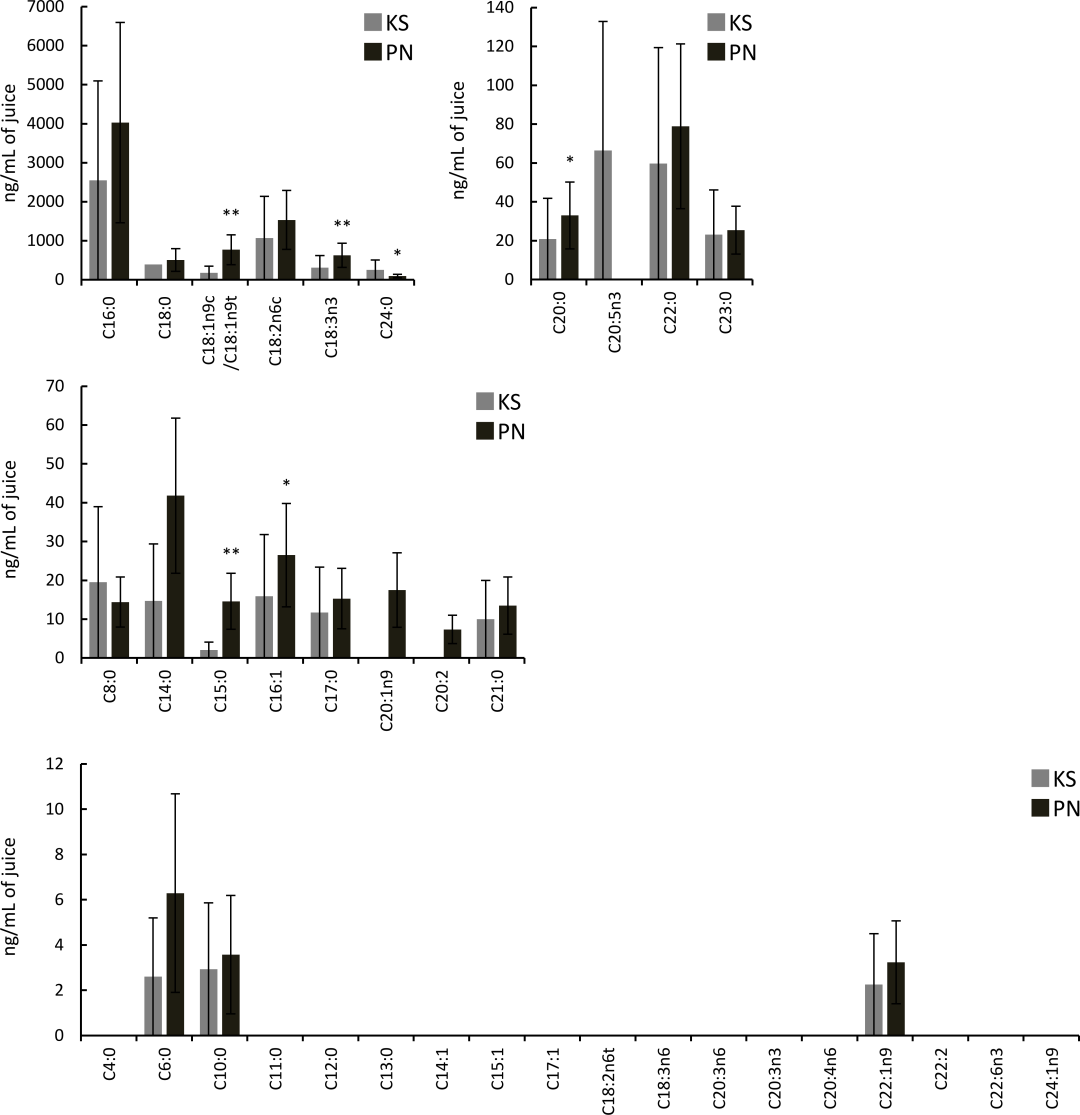
Fatty acids in Koshu and Pinot Noir juices. Data are shown as means ± standard deviation for five independent samples. KS, Koshu. PN, Pinot Noir. *p < 0.05 between Koshu and Pinot Noir juices. **p < 0.01 between Koshu and Pinot Noir juices.

The juices had extremely low fatty acid contents. Koshu juices contained 19 fatty acids, whereas Pinot Noir juices contained 20 fatty acids (Fig 2). C20:1n9 and C20:2 were detected in only Pinot Noir juices. C20:5n3 was detected in only Koshu juices. The contents of C15:0, C16:1, C18:1n9c/C18:1n9t, C18:3n3, and C20:0 in Pinot Noir juices were significantly higher than those in Koshu juices. In the same manner as berry skins, C24:0 content in Koshu juices was higher than that in Pinot Noir juices.

### Preliminary analysis of lipid components in Koshu skins

A representative total ion current chromatogram and the mass spectra obtained from 20 mg of Koshu skins are shown in S2 and S3 Figs, respectively. The peak with the highest intensity of in the mass spectra was detected at *m/z* 758.5681 (S3A Fig). A large ion peak was detected at *m/z* 496.3390 from peaks having high polarity with retention times of 10.53–11.99 min (S3B Fig) and a high-intensity peak at *m/z* 894.7533 was observed from peaks having low polarity with retention times of 18.01–23.98 min (S3C Fig). The main peaks were observed at the retention times of 10.79 min, 14.81 min, and 19.12 min in the mass spectra of *m/z* 496.3390, *m/z* 758.5681, and *m/z* 894.7533, respectively (S4 Fig). From the fragment patterns in the MS/MS spectra of these peaks, the peaks from *m/z* 496.3390, *m/z* 758.5681, and *m/z* 894.7533 were presumed to be protonated lysophosphatidylcholine (LPC) 16:0 (exact mass, 495.3325), protonated phosphatidylcholine (PC)34:2 (exact mass, 757.5622), and ammonium-ion-adducted triglyceride (TG) 54:7 (exact mass, 876.7207), respectively (S5 Fig). These results suggested that LPC 16:0, PC 34:2, and TG 54:7 were the major lipid components in Koshu skins.

### Reproducibility and linearity of lipid component quantification in Koshu skins

The reproducibility and the linearity of quantification of the main lipid components using our experimental methods were evaluated. The peak areas of each lipid in three independent experiments were stable even if skin volume used was changed (S7 Table). The CVs were from 1.5 to 6.2. Skin volume used and peak area showed positive correlations irrespective of lipid components (S6 Fig). In either lipid component, the coefficient of determination (r^2^) was higher than 0.996. Taken together, these results suggested that our experimental methods for lipid component profiling in berry skins had high reproducibility and linearity of lipid component quantification.

### Lipid components in Koshu and Pinot Noir skins

**Fig 3:**
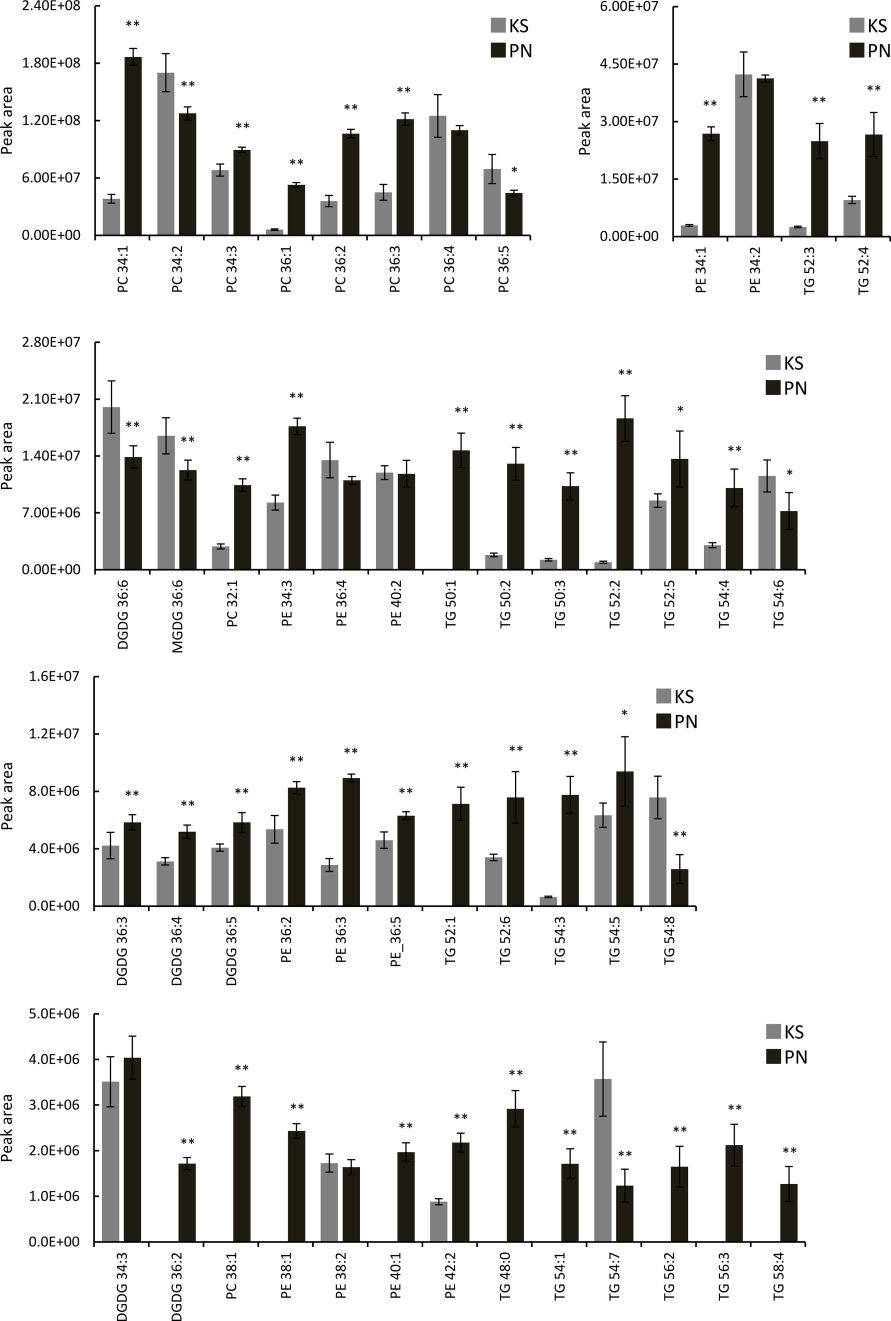
Lipid components in Koshu and Pinot Noir skins. Data are shown as means ± standard deviation for five independent samples. KS, Koshu. PN, Pinot Noir. *p < 0.05 between Koshu and Pinot Noir skins. **p < 0.01 between Koshu and Pinot Noir skins.

Forty-nine lipid components were identified from Koshu and Pinot Noir skins (Table 1). Specifically, there were six digalactosyldiacylglycerols (DGDGs), one monogalactosyldiacylglycerol (MGDG), 10 PCs, 12 phosphatidylethanolamines (PEs), and 20 TGs. The representative MS/MS spectra of each lipid class (DGDG 36:6, MGDG 36:6, PC 36:5, PE 34:2, and TG 54:6) are shown in S7 Fig. Then, a comparison of the lipid profiles of Koshu and Pinot Noir skins was performed (Fig 3). The contents of 36 of the 49 lipid components were significantly higher in Pinot Noir skins than Koshu skins. Specifically, strong peaks of DGDG 36:2, PC 38:1, PE 38:1, PE 40:1, TG 48:0, TG 50:1, TG 52:1, TG 54:1, TG 56:2, TG 56:3, and TG 58:4 were detected in Pinot Noir skins. In contrast, strong peaks of MGDG 36:6, DGDG 36:6, PC 34:2, PC 36:5, TG 54:6, TG 54:7, and TG 54:8 were observed in Koshu skins. These results suggested that Pinot Noir skins contained more lipid species whose alkyl chains have more than 18 carbon atoms than Koshu skins.

**Table 1:** Lipid components detected in Koshu and Pinot Noir skins.

Class	Compound	*m*/*z*	Ionization	Retention time (min)
DGDG	DGDG 34:3	932.630	[M+NH_4_]^+^	14.7
	DGDG 36:2	962.677	[M+NH_4_]^+^	15.4
	DGDG 36:3	960.662	[M+NH_4_]^+^	15.1
	DGDG 36:4	958.646	[M+NH_4_]^+^	14.7
	DGDG 36:5	956.631	[M+NH_4_]^+^	14.3
	DGDG 36:6	954.616	[M+NH_4_]^+^	14.0
MGDG	MGDG 36:6	792.561	[M+NH_4_]^+^	14.6
PC	PC 32:1	732.554	[M+H]^+^	14.8
	PC 34:1	760.585	[M+H]^+^	15.2
	PC 34:2	758.569	[M+H]^+^	14.8
	PC 34:3	756.554	[M+H]^+^	14.5
	PC 36:1	788.616	[M+H]^+^	15.8
	PC 36:2	786.600	[M+H]^+^	15.3
	PC 36:3	784.584	[M+H]^+^	14.8
	PC 36:4	782.568	[M+H]^+^	14.5
	PC 36:5	780.553	[M+H]^+^	14.1
	PC 38:1	816.647	[M+H]^+^	16.5
PE	PE 34:1	718.538	[M+H]^+^	15.4
	PE 34:2	716.522	[M+H]^+^	15.0
	PE 34:3	714.507	[M+H]^+^	14.6
	PE 36:2	744.554	[M+H]^+^	15.5
	PE 36:3	742.539	[M+H]^+^	15.0
	PE 36:4	740.523	[M+H]^+^	14.6
	PE 36:5	778.538	[M+(CH_3_)_2_CO+H-H_2_O]^+^	14.3
	PE 38:1	774.601	[M+H]^+^	16.4
	PE 38:2	772.585	[M+H]^+^	16.0
	PE 40:1	802.632	[M+H]^+^	16.9
	PE 40:2	800.617	[M+H]^+^	16.5
	PE 42:2	828.648	[M+H]^+^	17.0
TG	TG 48:0	824.770	[M+NH_4_]^+^	21.9
	TG 50:1	850.786	[M+NH_4_]^+^	21.7
	TG 50:2	848.771	[M+NH_4_]^+^	21.0
	TG 50:3	846.755	[M+NH_4_]^+^	20.5
	TG 52:1	878.817	[M+NH_4_]^+^	22.4
	TG 52:2	876.802	[M+NH_4_]^+^	21.6
	TG 52:3	874.786	[M+NH_4_]^+^	20.9
	TG 52:4	872.771	[M+NH_4_]^+^	20.3
	TG 52:5	870.755	[M+NH_4_]^+^	19.7
	TG 52:6	868.739	[M+NH_4_]^+^	19.2
	TG 54:1	906.848	[M+NH_4_]^+^	23.2
	TG 54:3	902.817	[M+NH_4_]^+^	21.5
	TG 54:4	900.802	[M+NH_4_]^+^	20.9
	TG 54:5	898.786	[M+NH_4_]^+^	20.2
	TG 54:6	896.770	[M+NH_4_]^+^	19.6
	TG 54:7	877.728	[M+H]^+^	19.1
	TG 54:8	892.739	[M+NH_4_]^+^	18.6
	TG 56:2	932.864	[M+NH_4_]^+^	23.1
	TG 56:3	930.848	[M+NH_4_]^+^	22.2
	TG 58:4	956.864	[M+NH_4_]^+^	22.2

### Number of double bonds in fatty acid molecule

**Fig 4:**
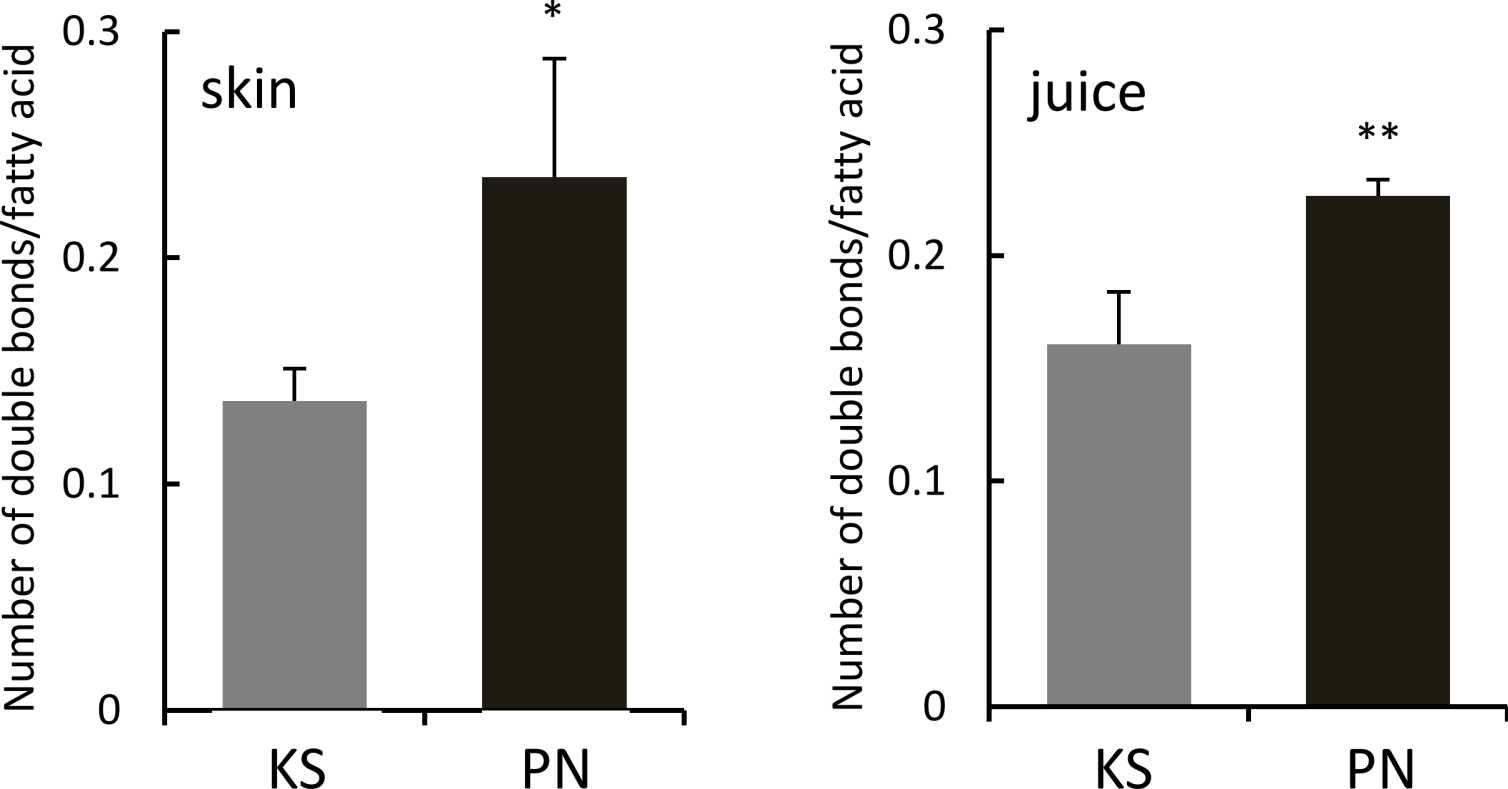
Number of double bonds in a fatty acid molecule in Koshu and Pinot Noir skins and juices. The number of double bonds in a fatty acid molecule in skin and juice was calculated from the data in Figs 1 and 2, respectively. Data are shown as means ± standard deviation for five independent samples. KS, Koshu. PN, Pinot Noir. *p < 0.05 between Koshu and Pinot Noir skins. **p < 0.01 between Koshu and Pinot Noir juices.

Lipid analysis of Koshu and Pinot Noir skins also demonstrated that lipid species containing double bonds existed abundantly in Koshu skins (Fig 3). Then, the number of double bonds in each fatty acid molecule in Koshu and Pinot Noir skins and juices was calculated from the data of Figs 1 and 2. The number of double bonds in a fatty acid molecule in Pinot Noir skins and juices was significantly larger than that in Koshu skins and juices (Fig 4).

### C18-fatty acid desaturation in Koshu and Pinot Noir skins

**Fig 5:**
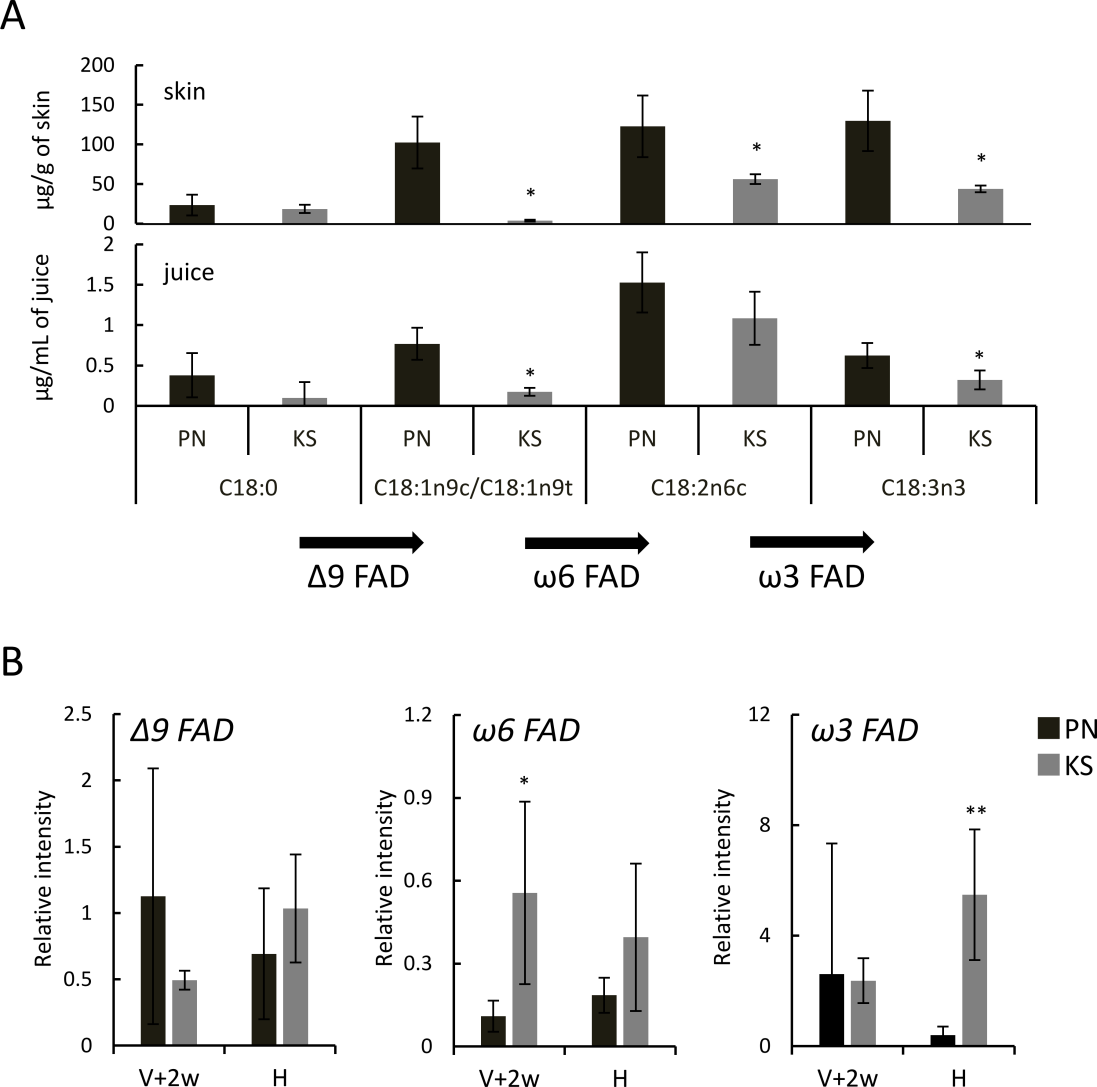
C18-fatty acid desaturation in Koshu and Pinot Noir berries. (A) C18-fatty acids in Pinot Noir and Koshu skins and juices. The contents were extracted from Figs 1 and 2, respectively. KS, Koshu. PN, Pinot Noir. *p < 0.05 between Koshu and Pinot Noir samples. Arrows demonstrate conversion by C18-fatty acid desaturases. Δ9 FAD, stearoyl-[acyl-carrier-protein] 9-desaturase. ω6 FAD, omega-6 fatty acid desaturase. ω3 FAD, omega-3 fatty acid desaturase. (B) Transcription of genes encoding C18-fatty acid desaturases in berry skins. Berry samples were collected at two weeks post véraison (V+2w) and at harvest (H). Total RNA was isolated from berry skins and subjected to real-time RT-PCR analysis. Data are shown as means ± standard deviation for five independent samples. *p < 0.05 between Koshu and Pinot Noir skins. **p < 0.01 between Koshu and Pinot Noir skins.

Fig 1 demonstrates that C18-fatty acids are one of the major components in *V*. *vinifera* berries, as reported previously [9]. Fig 5A shows the contents of C18-fatty acids (C18:0, C18:1n9c/C18:1n9t, C18:2n6c, and C18:3n3) in Koshu and Pinot Noir skins and juices, extracted from Figs 1 and 2. The contents of unsaturated C18-fatty acids (C18:1n9c/C18:1n9t, C18:2n6c, and C18:3n3) in Koshu berries were lower than those in Pinot Noir berries (Fig 5A).

For expression analysis of C18-fatty acid metabolism-related genes and genes encoding lipoxygenase (LOX) and hydroperoxide lyase (HPL) in the lipoxygenase pathway, we selected the members of them according to NIH genetic sequence database. However, some of them could not be analyzed by real-time RT-PCR due to less specificity of the primers and less expression in berry skins tested (data not shown). No difference in stearoyl-[acyl-carrier-protein] 9-desaturase gene expression was observed between Koshu and Pinot Noir skins at two weeks post véraison and at harvest (Fig 5B). The gene expression of omega-6 fatty acid desaturase and omega-3 fatty acid desaturase in Koshu skins was upregulated at two weeks post véraison and at harvest, respectively. These results suggested that the expression levels of C18-fatty acid desaturases might be one of the regulators of the accumulation of C18-unsaturated fatty acids in berry skins.

**Fig 6:**
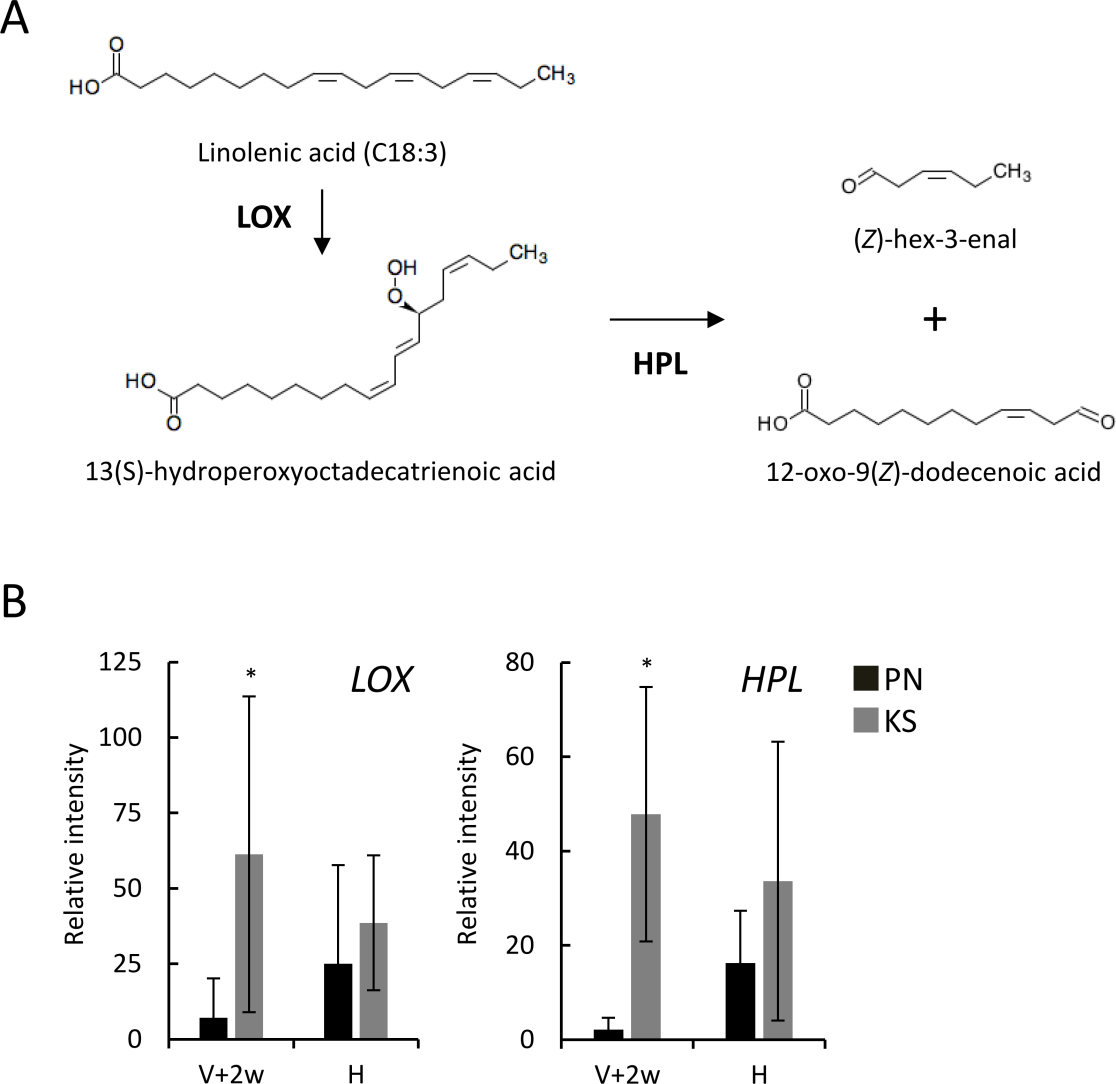
Expression of LOX and HPL genes in Koshu and Pinot Noir berries. (A) Conversion of C18:3 into (*Z*)-hex-3-enal via the LOX pathway. LOX, lipoxygenase. HPL, hydroperoxide lyase. (B) Transcription of genes encoding LOX and HPL in berry skins. Berry samples were collected at two weeks post véraison (V+2w) and at harvest (H). Total RNA was isolated from berry skins and subjected to real-time RT-PCR analysis. Data are shown as means ± standard deviation for five independent samples. *p < 0.05 between Koshu and Pinot Noir skins.

C18:3 is converted into hexanals, C6 aroma compounds, in plants [17]. Fig 6A shows the lipoxygenase pathway for the conversion of C18:3 into (*Z*)-hex-3-enal. The transcription of genes encoding LOX and HPL, the key enzymes in the pathway, was upregulated in Koshu skins at two weeks after véraison (Fig 6B). This result suggested that Koshu berries converted C18:3 into C6-aroma compounds and well accounted for the finding that C18:3n3 contents in Koshu skins and juices were lower than those in Pinot Noir skins and juices (Fig 5A) even if the gene expression of omega-3 fatty acid desaturase was upregulated (Fig 5B).

## Discussion

Plant lipidome research has seen an upsurge of interest. Although plants share almost the same lipid metabolism, plant lipidome is one of the tools for the discovery of unique lipids and oils in plants. Plant lipidome is expected to contribute to the application of unique plant lipids to human health and high-energy bioproducts [18]. In the present study, we uncovered differences in lipid compositions between Koshu and Pinot Noir berries cultivated under the same conditions by the comprehensive and comparative lipidome analysis. As lipid composition in grapevine organs is affected by environmental factors [19] and training systems [10], lipid compositions detected under different cultivation conditions in previous studies [14,15] cannot be compared with each other. In the present study, to optimize the experimental conditions, Koshu and Pinot Noir grapevines were separated by only 10 m in the same vineyard and trained to the Guyot-style system, although the traditional shelf style (overhead trellis) is generally used for the cultivation of Koshu [20]. These efforts to optimize the experimental design allowed us to perform comparative lipidome analysis of Koshu and Pinot Noir berries.

Fatty acid molecules in Pinot Noir berries had more double bonds than those in Koshu berries. In general, the increase in the number of double bonds in a fatty acid molecule is observed in plants growing in low-temperature regions [21]. Normal leaf development in plants growing in low-temperature condition would require increasing the production of trienoic fatty acid, for example C18:3, by fatty acid desaturation during chilling acclimation [22,23]. In C18-fatty acid metabolism, the contents of C18 unsaturated fatty acids in Pinot Noir berries were higher than those in Koshu berries. As C18:2 and C18:3 are the major components in plasma membrane [24], Pinot Noir berries might be able to adapt well to a cool climate by enhancing plasma membrane stability. Pinot Noir grapevines grow well in cool regions, whereas Koshu grapevines show preference for warm climate. As Pinot Noir and Koshu grapevines were cultivated under same field conditions in the present study, the difference in the level of fatty acid desaturation between the two cultivars might be a characteristic innate in the genome and not be acquired as a result of environmental conditions. Further studies employing comparative genomics to examine Pinot Noir [6] and Koshu genomes (Suzuki, personal communication) are necessary to prove the characteristic innate in their genome.

Koshu berries contained less C18:3 than Pinot Noir berries, although the gene expression of omega-3 fatty acid desaturase in Koshu skins was upregulated. In general, the upregulated expression of omega-3 fatty acid desaturase gene decreases C18:2 content and increases C18:3 content [22]. This contradiction may be due to the production of hexanals from C18:3 in Koshu berries. C18:3 is the precursor of (*Z*)-hex-3-enal [25]. The upregulation of omega-3 fatty acid desaturase expression enhances (*Z*)-hex-3-enal aroma in tomato fruit [26]. The conversion of C18:3 into C6-aroma compounds hexanals in plant is mediated by the LOX pathway [17]. Grape LOX family consists of 18 individual members and catalyzes oxygenation of polyunsaturated fatty acids [27]. Transcriptional patterns of each LOX genes were different specially and temporally. For example, *VvLOXA* was expressed abundantly in berry skins, while *VvLOXO* was predominantly expressed in seeds [27]. Modification of lipid profile in grape berries by LOX seems to be associated with berry softening. In Pinot Noir berries, PnLOXA was expressed at véraison and mediated membrane peroxidation, such as peroxisized monogalactosyl diacylglycerols and digalactosyl diacylglycerols on C18:3 chains, followed by berry softening [28]. These reports suggest that LOX is one of mediator during grape berry ripening. LOX and HPL gene transcription was upregulated during Koshu berry ripening, although all of the members could not be analyzed due to less specificity of PCR primers tested. The enhanced LOX and HPL expression may decrease C18:3 content and conversely increase (Z)-hex-3-enal in Koshu skins. (*Z*)-hex-3-enal is converted into (*E*)-hex-2-enal (*trans*-2-hexanal) by isomerases [25]. 3-Mercaptohexan-1-ol is a volatile thiol and a key contributor to the distinct passion fruit-like odor in Koshu wines but not Pinot Noir wines. The precursors of 3-mercaptohexan-1-ol are synthesized from glutathione and (*E*)-hex-2-enal by glutathione-*S*-transferases during berry ripening [29]. Thus, the loss of C18:3 in Koshu berries by the LOX pathway at the end of ripening may be correlated with the synthesis of C6-aroma compounds and the precursors of 3-mercaptohexan-1-ol.

We demonstrated that Koshu lipid profiles were completely different from Pinot Noir lipid profiles. The lipid profiles obtained from grape lipidome might contribute to the alteration of aroma profiles in Koshu wines. Berries cultivated in cold regions showed higher linoleic acid content than berries cultivated in warm regions [30]. The high C18:2 levels in juices were related to low fatty acid ethyl ester formation in wine yeasts [30]. Further investigations of both viticultural techniques and the selection of areas for Koshu cultivation are required for improving Koshu wine aromas through the alteration of fatty acid profiles in Koshu berries.

As there is a strong relationship between the heat requirements of grapevine cultivars and the level of fatty acid desaturation [30], grapevine lipidome may also contribute to the improvement of breeding strategies of cold-tolerant grapevines. Grapevine cultivated in cool areas is frequently exposed to temperatures below 0°C in winter and the low temperature affects the growth and development of grapevine organs, such as dormant buds, leaves, and flowers, in the next growing season [31]. The production of trienoic fatty acids by fatty acid desaturase enhances cold tolerance in plants [22]. Although C-repeat-binding factors that activate the transcription of cold-responsive genes play a critical role in cold acclimation in grapevines [32], fatty acid desaturase genes may also serve as a breeding target of cold-tolerant grapevines to enhance adaptation to a chilly environment.

## Supporting information

S1 Fig Chromatograms of supplemented fatty acids in Koshu juice and skin samples. (TIF)

S2 Fig Total ion current chromatogram of lipid components in Koshu skins. (TIF)

S3 Fig Mass spectra of lipid components in Koshu skins. (A) Retention time of 5.00–29.97 min. (B) Retention time of 10.53–11.99 min. (C) Retention time of 18.01–23.98 min.
(TIF)

S4 Fig Mass spectra of predominant lipid components in Koshu skins. (A) *m*/*z* 496.3390. (B) *m*/*z* 758.5681. (C) *m*/*z* 894.7533.
(TIF)

S5 Fig MS/MS spectra and precursor ions of predominant lipid components in Koshu skins. (A) Protonated lysophosphatidylcholine 16:0, *m*/*z* 496.3390, [M+H]^+^. (B) Protonated phosphatidylcholine 34:2, *m*/*z* 758.5681, [M+H]^+^. (C) Ammonium-ion-adducted triglyceride 54:7, *m*/*z* 894.7533, [M+NH_4_]^+^. Suggested structures are shown in the graphs.
(TIF)

S6 Fig Correlations between volumes of skin samples and peak areas obtained by LC-MS. (A) LPC 16:0. (B) PC 34:2. (C) TG 54:7.
(TIF)

S7 Fig Representative MS/MS spectra of each lipid class detected in Koshu skins. (A) DGDG 36:6. (B) MGDG 36:6. (C) PC 36:5. (D) PE 34:2. (E) TG 54:6.
(TIF)

S1 Table Conditions for multiple reaction monitoring in GC-MS/MS for fatty acids. (DOCX)

S2 Table Peaks of fatty acids in fatty acid recovery test. (DOCX)

S3 Table List of fatty acids in F.A.M.E. Mix C4-C24. (DOCX)

S4 Table Lower limits of quantification of fatty acids in juices. (DOCX)

S5 Table Lower limits of quantification of fatty acids in skins. (DOCX)

S6 Table Recovered amounts, recovery rates, and coefficients of variation (CVs) for juice and skin samples supplemented with fatty acids. (DOCX)

S7 Table Reproducibility and linearity of peak areas of main lipid components in Koshu skins. (DOCX)
