# Supplementary figures and images for: Comprehensive and comparative lipidome analysis of Vitis vinifera L. cv. Pinot Noir and Japanese indigenous V. vinifera L. cv. Koshu grape berries
Source: PLoS One. 2017 Oct 20;12(10):e0186952. doi: 10.1371/journal.pone.0186952 (PMC5650187; doi:10.1371/journal.pone.0186952)

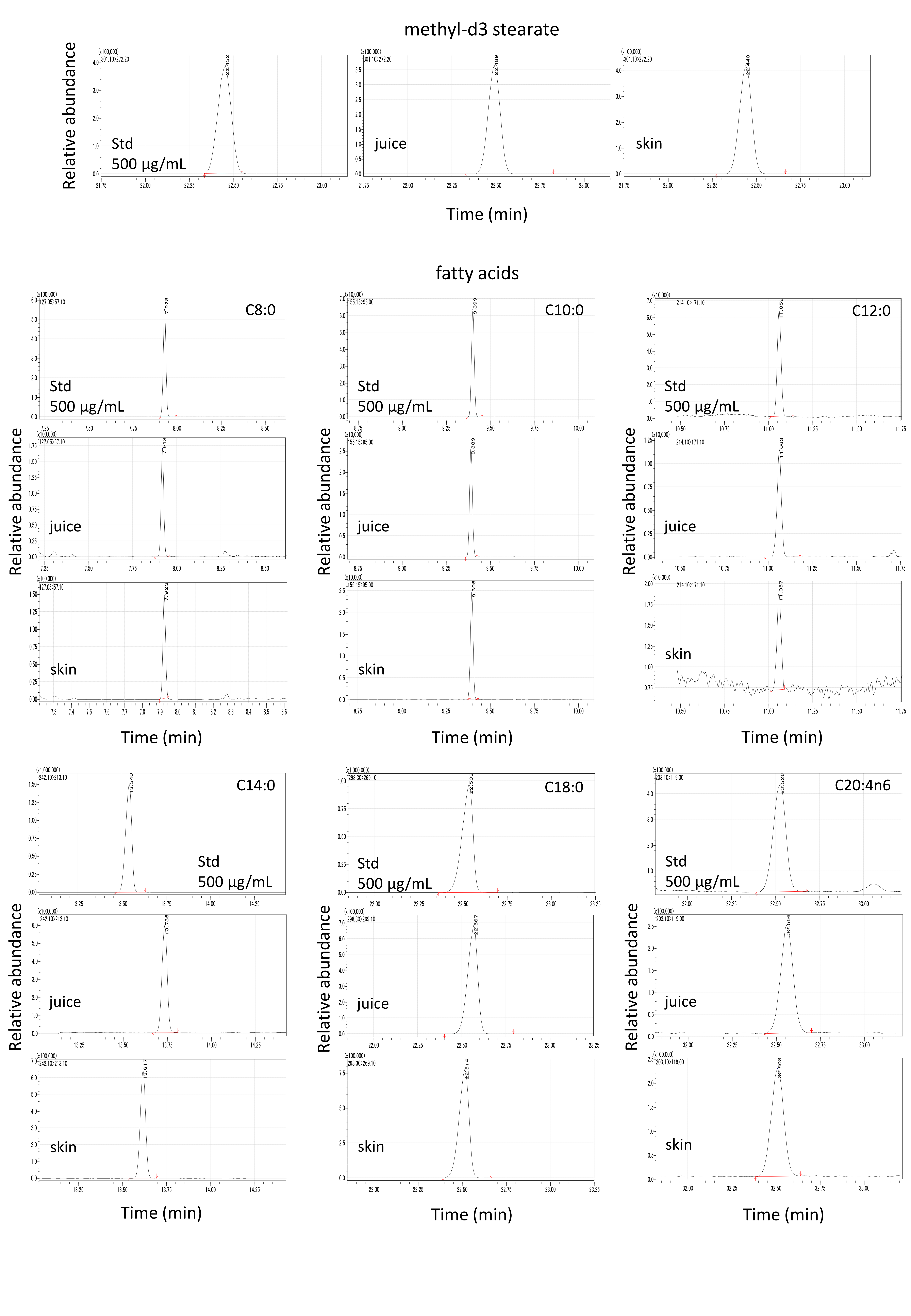

Supplement: S1 Fig — (TIF) [file pone.0186952.s001.tif]

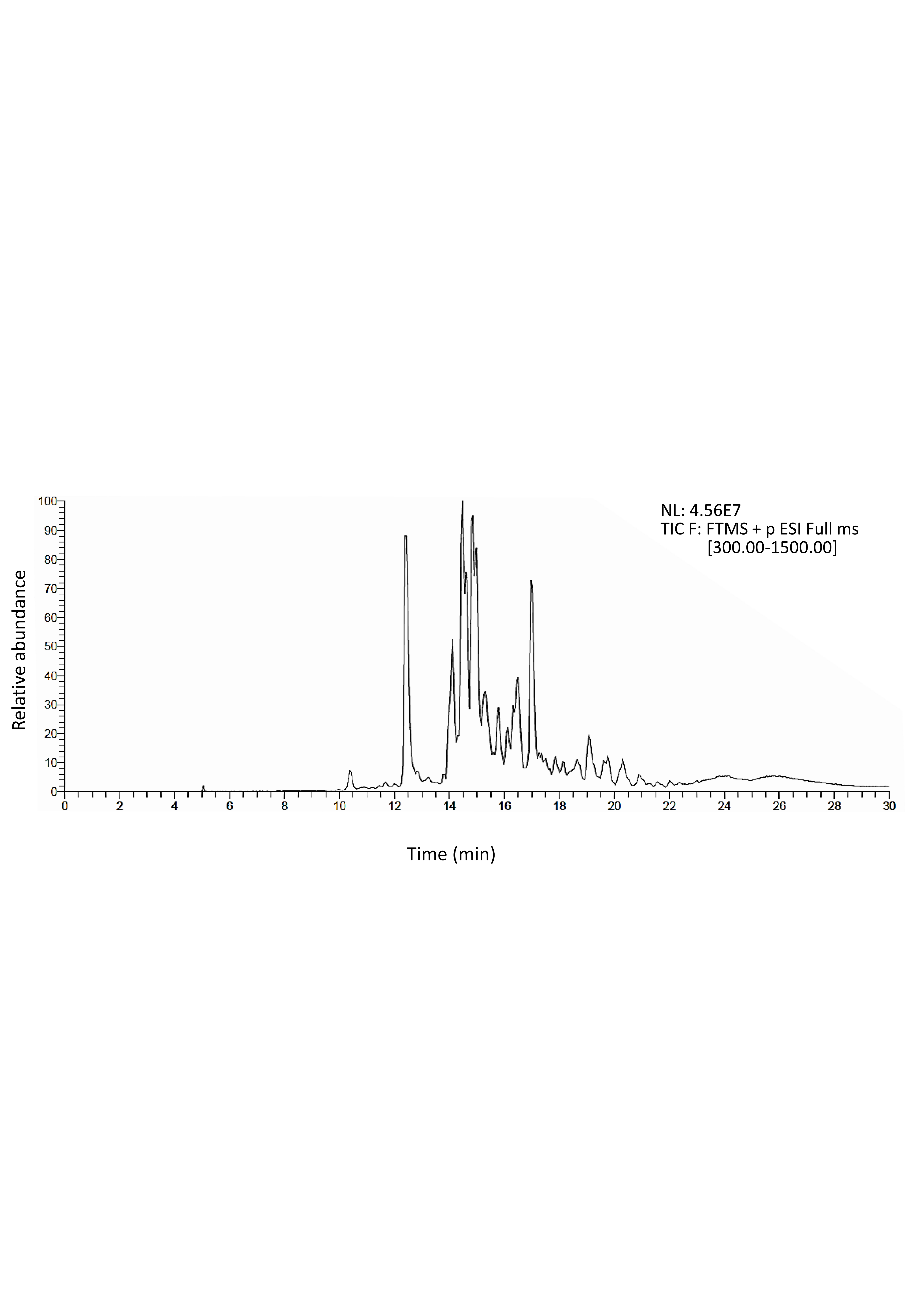

Supplement: S2 Fig — (TIF) [file pone.0186952.s002.tif]

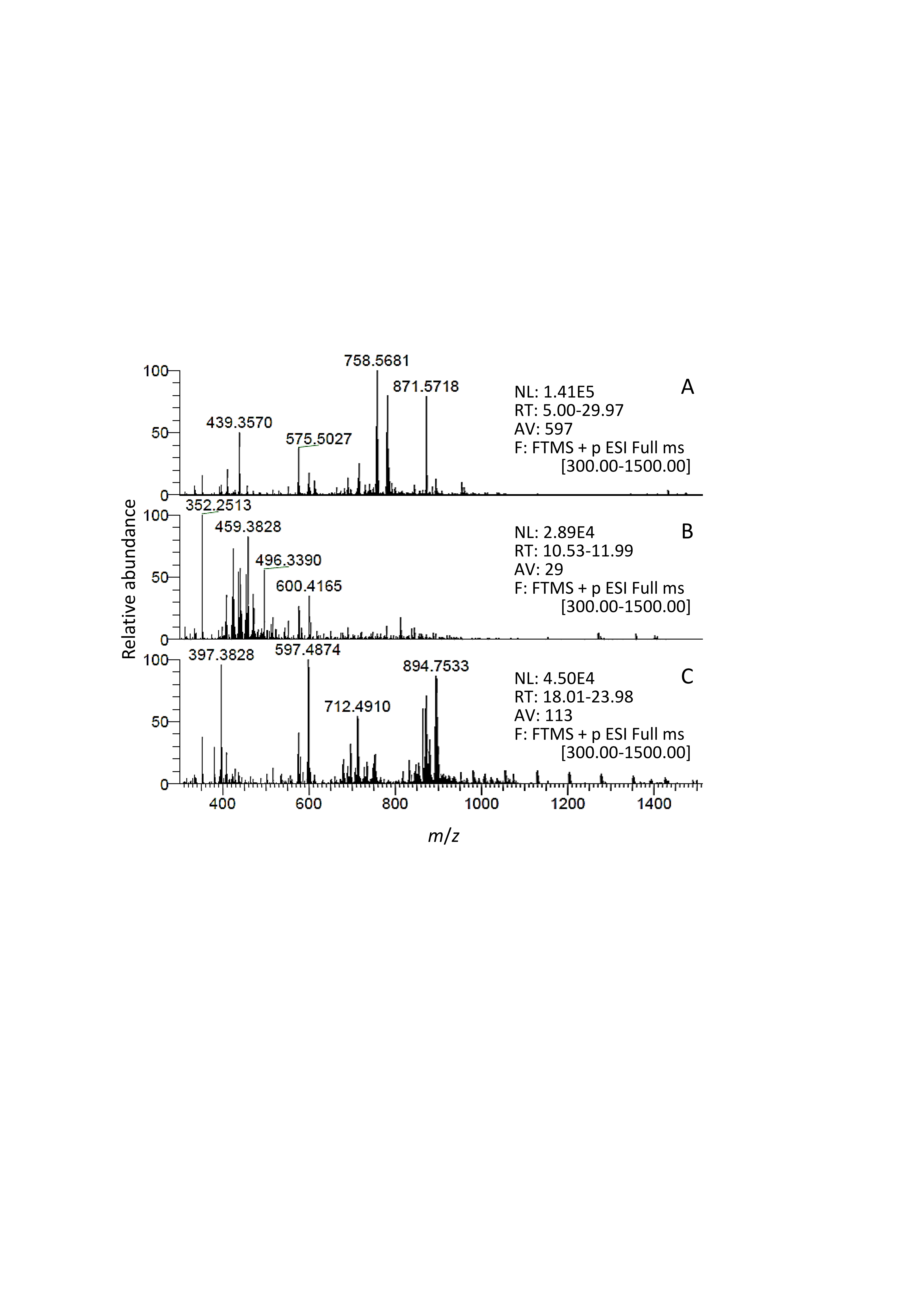

Supplement: S3 Fig — (A) Retention time of 5.00–29.97 min. (B) Retention time of 10.53–11.99 min. (C) Retention time of 18.01–23.98 min. (TIF) [file pone.0186952.s003.tif]

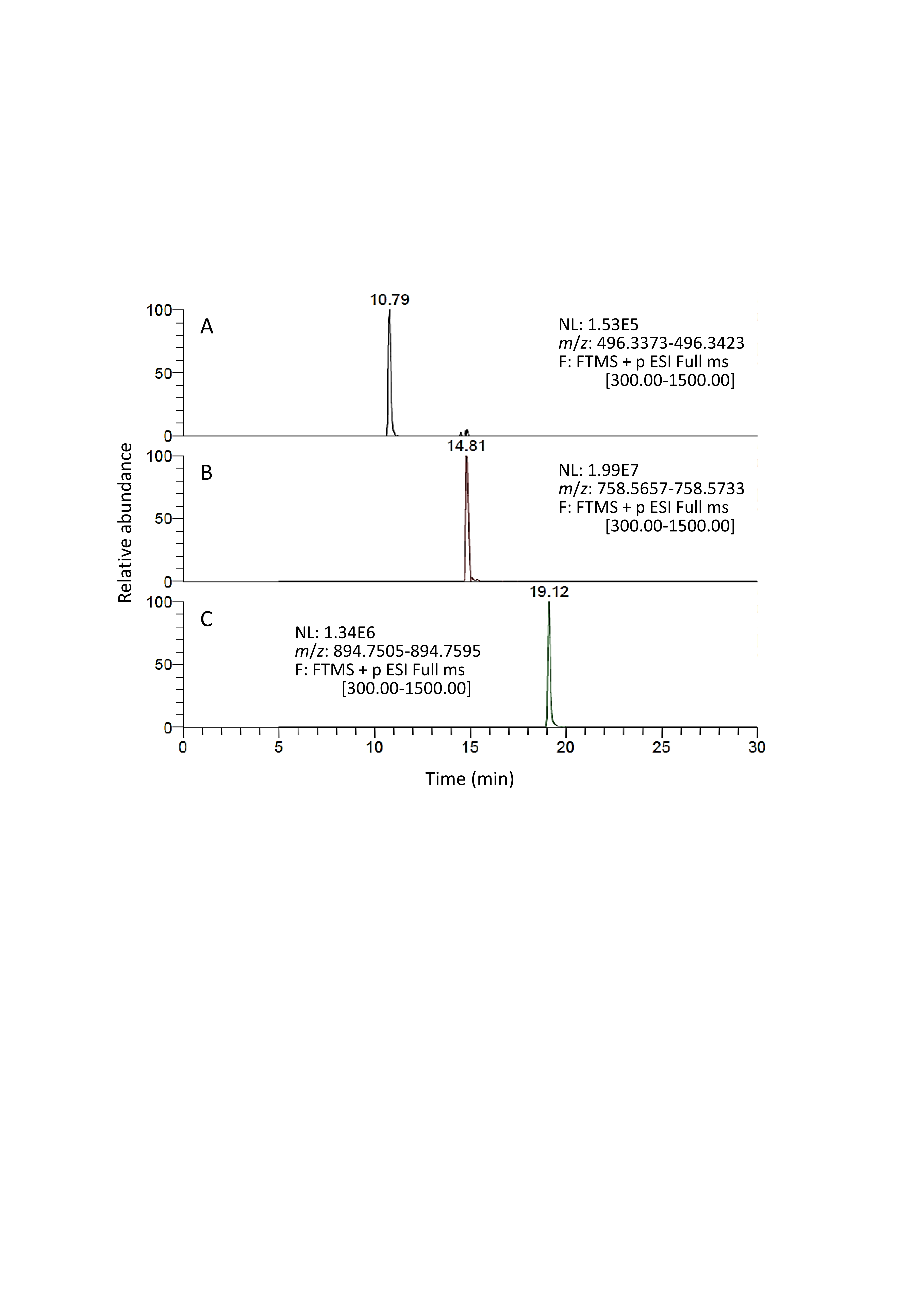

Supplement: S4 Fig — (A) m/z 496.3390. (B) m/z 758.5681. (C) m/z 894.7533. (TIF) [file pone.0186952.s004.tif]

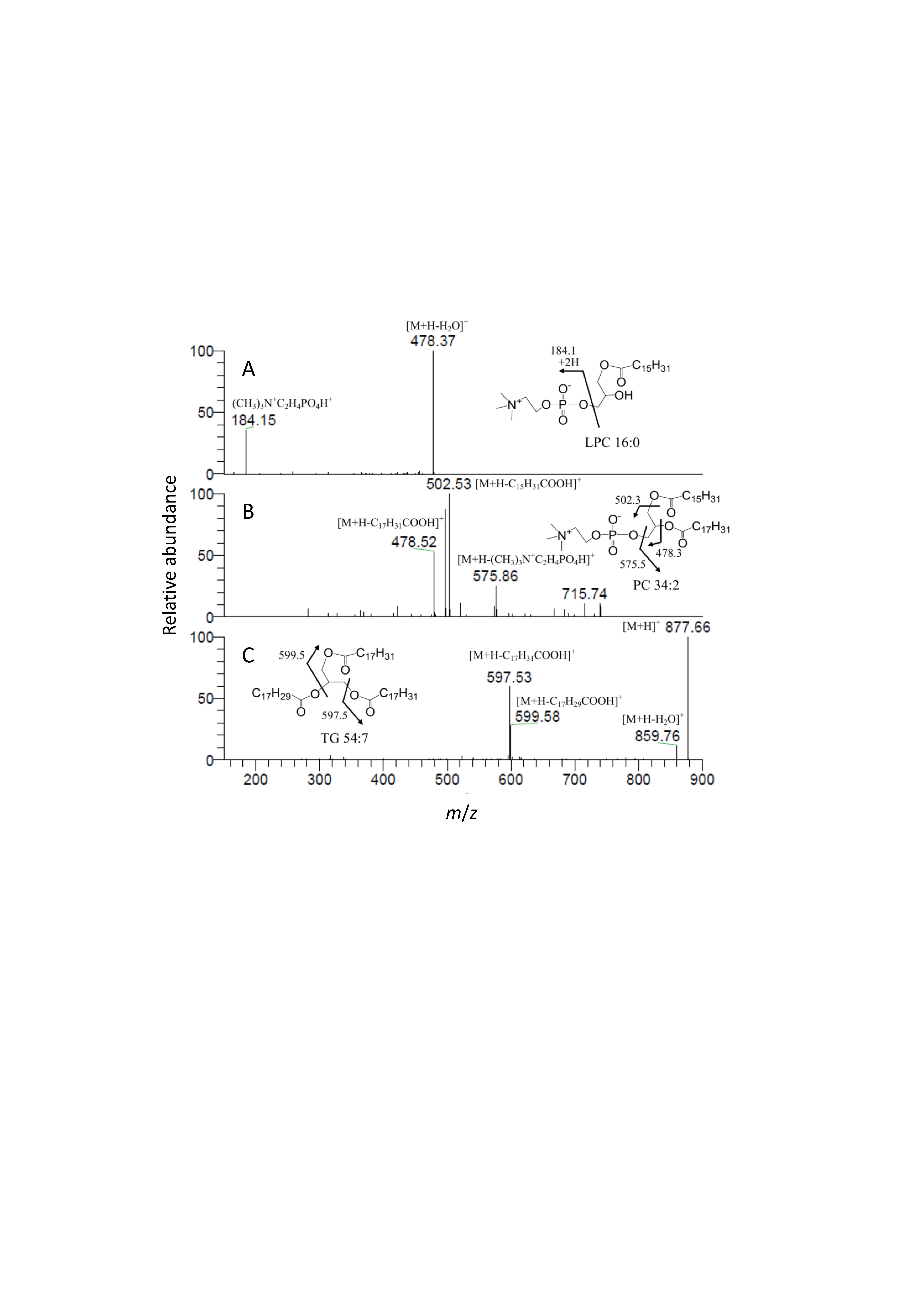

Supplement: S5 Fig — (A) Protonated lysophosphatidylcholine 16:0, m/z 496.3390, [M+H]+. (B) Protonated phosphatidylcholine 34:2, m/z 758.5681, [M+H]+. (C) Ammonium-ion-adducted triglyceride 54:7, m/z 894.7533, [M+NH4]+. Suggested structures are shown in the graphs. (TIF) [file pone.0186952.s005.tif]

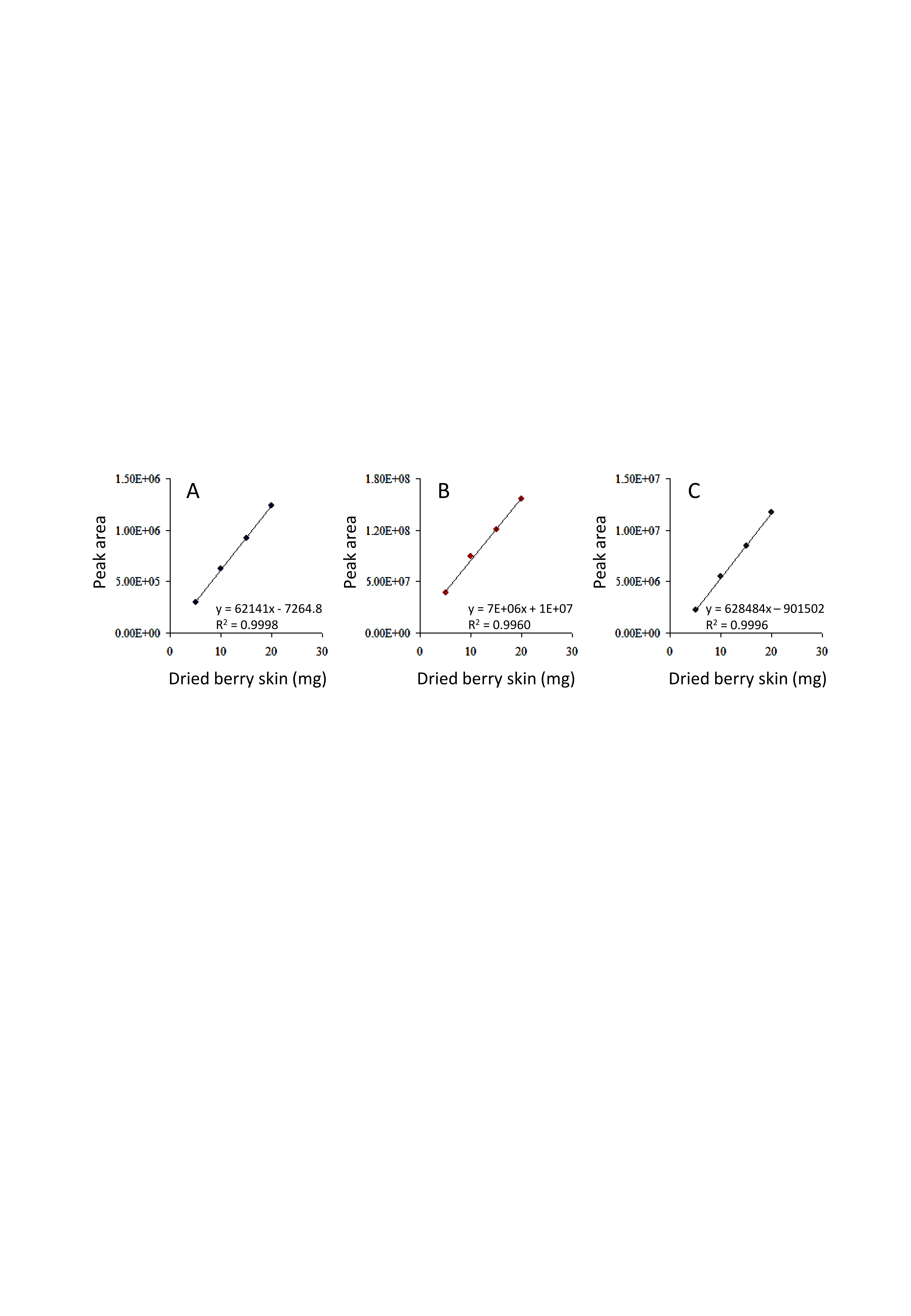

Supplement: S6 Fig — (A) LPC 16:0. (B) PC 34:2. (C) TG 54:7. (TIF) [file pone.0186952.s006.tif]

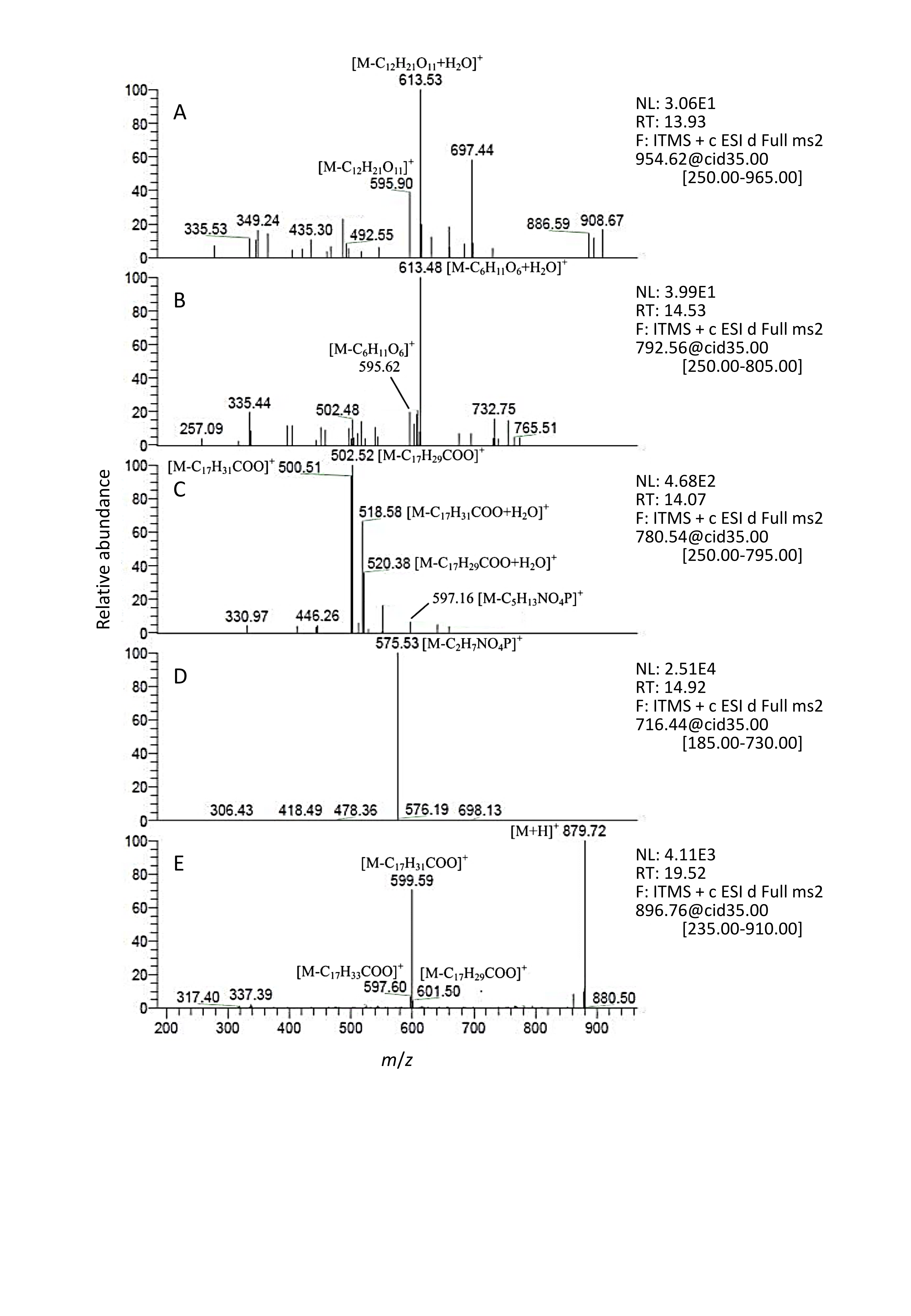

Supplement: S7 Fig — (A) DGDG 36:6. (B) MGDG 36:6. (C) PC 36:5. (D) PE 34:2. (E) TG 54:6. (TIF) [file pone.0186952.s007.tif]
