# Supplementary material for: Comprehensive and comparative lipidome analysis of Vitis vinifera L. cv. Pinot Noir and Japanese indigenous V. vinifera L. cv. Koshu grape berries
Source: PLoS One. 2017 Oct 20;12(10):e0186952. doi: 10.1371/journal.pone.0186952 (PMC5650187; doi:10.1371/journal.pone.0186952)
Supplement: S1 Table — (DOCX) [file pone.0186952.s008.docx]

| **S1 Table.** Conditions for multiple reaction monitoring in GC-MS/MS for fatty acids. | | | | | | |
| --- | --- | --- | --- | --- | --- | --- |
| Compound | Start time (min) | End time (min) | Detected ion 1  (Quantitative) | Collision energy 1  (V) | Detected ion 2  (Qualitative) | Collision energy 2  (V) |
| Butyric Acid Methyl Ester | 3.5 | 10.48 | 87.10>55.10 | 12 | 74.05>43.00 | 12 |
| Caproic Acid Methyl Ester | 3.5 | 10.48 | 99.05>71.10 | 8 | 99.05>43.00 | 8 |
| Caproic Acid Methyl Ester-d_11_ | 3.5 | 10.48 | 110.05>82.10 | 8 | 110.05>50.10 | 8 |
| Caprylic Acid Methyl Ester | 3.5 | 10.48 | 127.05>57.10 | 17 | 127.05>43.00 | 17 |
| Capric Acid Methyl Ester | 3.5 | 10.48 | 155.15>95.00 | 8 | 155.15>85.10 | 8 |
| Undecanoic Acid Methyl Ester | 3.5 | 10.48 | 169.15>109.10 | 8 | 169.15>95.00 | 8 |
| Undecanoic Acid Methyl Ester | 10.48 | 14.63 | 169.15>109.10 | 8 | 169.15>95.00 | 8 |
| Lauric Acid Methyl Ester | 10.48 | 14.63 | 214.10>171.10 | 8 | 214.10>157.10 | 8 |
| Lauric Acid Methyl Ester-d_3_ | 10.48 | 14.63 | 217.10>174.10 | 8 | 217.10>160.10 | 8 |
| Tridecanoic Acid Methyl Ester | 10.48 | 14.63 | 228.10>171.10 | 8 | 228.10>185.00 | 8 |
| Myristic Acid Methyl Ester | 10.48 | 14.63 | 242.10>213.10 | 8 | 242.10>199.10 | 8 |
| Myristic Acid Methyl Ester-d_27_ | 10.48 | 14.63 | 269.10>219.10 | 8 | 269.10>203.10 | 8 |
| Myristoleic Acid Methyl Ester | 10.48 | 14.63 | 240.00>197.10 | 10 | 240.00>141.10 | 10 |
| Pentadecanoic Acid Methyl Ester | 14.63 | 21.47 | 256.10>213.10 | 8 | 256.10>199.10 | 8 |
| *cis*-10-Pentadecenoic Acid Methyl Ester | 14.63 | 21.47 | 254.10>211.10 | 9 | 254.10>171.10 | 9 |
| Palmitic Acid Methyl Ester | 14.63 | 21.47 | 270.10>199.10 | 8 | 270.10>241.10 | 8 |
| Palmitoleic Acid Methyl Ester | 14.63 | 21.47 | 268.20>141.10 | 13 | 268.20>169.10 | 13 |
| Heptadecanoic Acid Methyl Ester | 14.63 | 21.47 | 284.30>255.20 | 8 | 284.30>199.20 | 8 |
| *cis*-10-Heptadecenoic Acid Methyl Ester | 14.63 | 21.47 | 282.20>199.10 | 13 | 282.20>165.10 | 13 |
| Myristoleic Acid Methyl Ester | 14.63 | 21.47 | 240.00>197.10 | 10 | 240.00>141.10 | 10 |
| Stearic Acid Methyl Ester | 21.47 | 27.62 | 298.30>269.10 | 8 | 298.30>213.10 | 8 |
| Stearic Acid Methyl Ester-d_3_ | 21.47 | 27.62 | 301.10>272.20 | 8 | 301.10>258.20 | 8 |
| Oleic Acid Methyl Ester and Elaidic Acid Methyl Ester | 21.47 | 27.62 | 296.20>253.20 | 8 | 296.20>213.10 | 8 |
| Linoleic Acid Methyl Ester and Linolelaidic Acid Methyl Ester | 21.47 | 27.62 | 294.30>150.10 | 13 | 294.30>110.10 | 13 |
| γ-Linolenic Acid Methyl Ester | 21.47 | 27.62 | 292.10>161.10 | 17 | 292.10>121.10 | 17 |
| α-Linolenic Acid Methyl Ester | 21.47 | 27.62 | 291.95>175.10 | 12 | 291.95>135.10 | 12 |
| *cis*-10-Heptadecenoic Acid Methyl Ester | 21.47 | 27.62 | 282.20>199.10 | 13 | 282.20>165.10 | 13 |
| Arachidic Acid Methyl Ester | 27.62 | 33.5 | 326.20>283.10 | 10 | 326.20>241.10 | 10 |
| *cis*-11-Eicosenoic Acid Methyl Ester | 27.62 | 33.5 | 324.30>253.10 | 10 | 324.30>141.10 | 10 |
| *cis*-11,14-Eicosadienoic Acid Methyl Ester | 27.62 | 33.5 | 322.20>290.10 | 7 | 322.20>138.10 | 7 |
| *cis*-8,11,14-Eicosatrienoic Acid Methyl Ester | 27.62 | 33.5 | 320.15>150.10 | 8 | 320.15>121.10 | 8 |
| Heneicosanoic Acid Methyl Ester | 27.62 | 33.5 | 340.20>185.10 | 8 | 340.20>255.10 | 8 |
| Arachidonic Acid Methyl Ester | 27.62 | 33.5 | 203.10>133.00 | 10 | 203.10>119.00 | 10 |
| α-Linolenic Acid Methyl Ester | 27.62 | 33.5 | 291.95>175.10 | 12 | 291.95>135.10 | 12 |
| *cis*-11,14,17-Eicosatrienoic Acid Methyl Ester | 33.5 | 40.7 | 320.00>149.10 | 10 | 320.00>191.10 | 10 |
| *cis*-5,8,11,14,17-Eicosapentaenoic Acid Methyl Ester | 33.5 | 40.7 | 215.30>145.10 | 12 | 215.30>159.10 | 12 |
| Behenic Acid Methyl Ester | 33.5 | 40.7 | 354.30>255.10 | 10 | 354.30>311.10 | 10 |
| Erucic Acid Methyl Ester | 33.5 | 40.7 | 320.10>277.10 | 10 | 320.10>221.10 | 10 |
| *cis*-13,16-Docosadienoic Acid Methyl Ester | 33.5 | 40.7 | 350.10>318.10 | 8 | 350.10>124.10 | 8 |
| Tricosanoic Acid Methyl Ester | 33.5 | 40.7 | 368.30>339.10 | 10 | 368.30>255.10 | 10 |
| Lignoceric Acid Methyl Ester | 40.7 | 48 | 382.30>353.10 | 10 | 382.30>283.10 | 10 |
| *cis*-4,7,10,13,16,19-Docosahexaenoic Acid Methyl Ester | 40.7 | 48 | 159.20>129.10 | 22.5 | 159.20>117.10 | 22.5 |
| Nervonic Acid Methyl Ester | 40.7 | 48 | 348.20>305.10 | 12 | 348.20>249.10 | 12 |
| Tricosanoic Acid Methyl ester | 40.7 | 48 | 368.30>339.10 | 10 | 368.30>255.10 | 10 |
