# Supplementary material for: Comprehensive and comparative lipidome analysis of Vitis vinifera L. cv. Pinot Noir and Japanese indigenous V. vinifera L. cv. Koshu grape berries
Source: PLoS One. 2017 Oct 20;12(10):e0186952. doi: 10.1371/journal.pone.0186952 (PMC5650187; doi:10.1371/journal.pone.0186952)
Supplement: S2 Table — (DOCX) [file pone.0186952.s009.docx]

| **S2 Table.** Peaks of fatty acids in fatty acid recovery test. | | |
| --- | --- | --- |
| Compound | Detected ion (*m/z*) | Retention time (min) |
| Caprylic Acid (C8:0) | 127.05>57.10 | 7.9 |
| Capric Acid (C10:0) | 155.15>95.00 | 9.4 |
| Lauric Acid (C12:0) | 214.10>171.10 | 11.1 |
| Myristic Acid (C14:0) | 242.10>213.10 | 13.5 |
| Stearic Acid (C18:0) | 298.30>269.10 | 22.5 |
| Arachidonic Acid (C20:4n6) | 203.10>133.00 | 32.5 |
