# Supplementary material for: Comprehensive and comparative lipidome analysis of Vitis vinifera L. cv. Pinot Noir and Japanese indigenous V. vinifera L. cv. Koshu grape berries
Source: PLoS One. 2017 Oct 20;12(10):e0186952. doi: 10.1371/journal.pone.0186952 (PMC5650187; doi:10.1371/journal.pone.0186952)
Supplement: S3 Table — (DOCX) [file pone.0186952.s010.docx]

| **S3 Table.** List of fatty acids in F.A.M.E. Mix C4-C24. | | | | | |
| --- | --- | --- | --- | --- | --- |
| No. | Compound | Detected compound in F.A.M.E Mix | Formula | ChEBI ID | Content  (%) |
| 1 | Butyric acid | Butyric acid methyl ester | C4:0 | CHEBI:30772 | 4 |
| 2 | Caproic acid | Caproic acid methyl ester | C6:0 | CHEBI:30776 | 4 |
| 3 | Caprylic acid | Caprylic acid methyl ester | C8:0 | CHEBI:28837 | 4 |
| 4 | Capric acid | Capric acid methyl ester | C10:0 | CHEBI:30813 | 4 |
| 5 | Undecanoic acid | Undecanoic acid methyl ester | C11:0 | CHEBI:32368 | 2 |
| 6 | Lauric acid | Lauric acid methyl ester | C12:0 | CHEBI:30805 | 4 |
| 7 | Tridecanoic acid | Tridecanoic acid methyl ester | C13:0 | CHEBI:45919 | 2 |
| 8 | Myristic acid | Myristic acid methyl ester | C14:0 | CHEBI:28875 | 4 |
| 9 | Myristoleic acid | Myristoleic acid methyl ester | C14:1 | CHEBI:27781 | 2 |
| 10 | Pentadecanoic acid | Pentadecanoic acid methyl ester | C15:0 | CHEBI:42504 | 2 |
| 11 | *cis*-10-Pentadecenoic acid | *cis*-10-Pentadecenoic acid methyl ester | C15:1 | CHEBI:75089 | 2 |
| 12 | Palmitic acid | Palmitic acid methyl ester | C16:0 | CHEBI:15756 | 6 |
| 13 | Palmitoleic acid | Palmitoleic acid methyl ester | C16:1 | CHEBI:28716 | 2 |
| 14 | Heptadecanoic acid | Heptadecanoic acid methyl ester | C17:0 | CHEBI:32365 | 2 |
| 15 | *cis*-10-Heptadecenoic acid | *cis*-10-Heptadecenoic acid methyl ester | C17:1 | CHEBI:75094 | 2 |
| 16 | Stearic acid | Stearic acid methyl ester | C18:0 | CHEBI:28842 | 4 |
| 17 | Oleic acid | Oleic acid methyl ester | C18:1n9c | CHEBI:16196 | 4 |
| 18 | Elaidic acid | Elaidic acid methyl ester | C18:1n9t | CHEBI:27997 | 2 |
| 19 | Linoleic acid | Linoleic acid methyl ester | C18:2n6c | CHEBI:17351 | 2 |
| 20 | Linolelaidic acid | Linolelaidic acid methyl ester | C18:2n6t | CHEBI:76215 | 2 |
| 21 | γ-Linolenic acid | γ-Linolenic acid methyl ester | C18:3n6 | CHEBI:28661 | 2 |
| 22 | α-Linolenic acid | α-Linolenic acid methyl ester | C18:3n3 | CHEBI:27432 | 2 |
| 23 | Arachidic acid | Arachidic acid methyl ester | C20:0 | CHEBI:28822 | 4 |
| 24 | *cis*-11-Eicosenoic acid | *cis*-11-Eicosenoic acid methyl ester | C20:1n9 | CHEBI:32425 | 2 |
| 25 | *cis*-11,14-Eicosadienoic acid | *cis*-11,14-Eicosadienoic acid methyl ester | C20:2 | CHEBI:73731 | 2 |
| 26 | *cis*-8,11,14-Eicosatrienoic acid | *cis*-8,11,14-Eicosatrienoic acid methyl ester | C20:3n6 | CHEBI:53486 | 2 |
| 27 | *cis*-11,14,17-Eicosatrienoic acid | *cis*-11,14,17-Eicosatrienoic acid methyl ester | C20:3n3 | CHEBI:53460 | 2 |
| 28 | Arachidonic acid | Arachidonic acid methyl ester | C20:4n6 | CHEBI:15843 | 2 |
| 29 | *cis*-5,8,11,14,17-Eicosapentaenoic acid | *cis*-5,8,11,14,17-Eicosapentaenoic acid methyl ester | C20:5n3 | CHEBI:28364 | 2 |
| 30 | Heneicosanoic acid | Heneicosanoic acid methyl ester | C21:0 | CHEBI:39248 | 2 |
| 31 | Behenic acid | Behenic acid methyl ester | C22:0 | CHEBI:28941 | 4 |
| 32 | Erucic acid | Erucic acid methyl ester | C22:1n9 | CHEBI:28792 | 2 |
| 33 | *cis*-13,16-Docosadienoic acid | *cis*-13,16-Docosadienoic acid methyl ester | C22:2 | CHEBI:75117 | 2 |
| 34 | *cis*-4,7,10,13,16,19-Docosahexaenoic acid | *cis*-4,7,10,13,16,19-Docosahexaenoic acid methyl ester | C22:6n3 | CHEBI:28125 | 2 |
| 35 | Tricosanoic acid | Tricosanoic acid methyl ester | C23:0 | CHEBI:42394 | 2 |
| 36 | Lignoceric acid | Lignoceric acid methyl ester | C24:0 | CHEBI:28866 | 4 |
| 37 | Nervonic acid | Nervonic acid methyl ester | C24:1n9 | CHEBI:44247 | 2 |
