# Supplementary material for: Comprehensive and comparative lipidome analysis of Vitis vinifera L. cv. Pinot Noir and Japanese indigenous V. vinifera L. cv. Koshu grape berries
Source: PLoS One. 2017 Oct 20;12(10):e0186952. doi: 10.1371/journal.pone.0186952 (PMC5650187; doi:10.1371/journal.pone.0186952)
Supplement: S4 Table — (DOCX) [file pone.0186952.s011.docx]

| **S4 Table.** Lower limits of quantification of fatty acids in juices. | | | |
| --- | --- | --- | --- |
| Compound | Concentration for calibration curve 1  (µg/mL) | Concentration for calibration curve 2  (µg/mL) | Lower limit  (ng/mL) |
| C4:0 | 0.25-250 | 0.25-1,000 | 1 |
| C6:0 | 1-250 | 1-1,000 | 4 |
| C8:0 | 0.25-250 | 0.25-1,000 | 1 |
| C10:0 | 1-250 | 1-1,000 | 4 |
| C11:0 | 5-250 | 5-1,000 | 10 |
| C12:0 | 1-250 | 1-1,000 | 4 |
| C13:0 | 1-250 | 1-5,000 | 6 |
| C14:0 | 5-250 | 5-5,000 | 20 |
| C14:1 | 0.25-100 | 0.25-1,000 | 0.5 |
| C15:0 | 0.25-100 | 0.25-1,000 | 1 |
| C15:1 | 1-100 | 1-1,000 | 2 |
| C16:0 | 0.25-100 | 0.25-1,000 | 0.5 |
| C16:1 | 1-100 | 1-1,000 | 2 |
| C17:0 | 1-100 | 1-1,000 | 2 |
| C17:1 | 0.25-100 | 0.25-1,000 | 0.5 |
| C18:0 | 5-100 | 5-1,000 | 10 |
| C18:1n9c/C18:1n9t | 1-100 | 1-1,000 | 6 |
| C18:2n6c | 1-100 | 1-1,000 | 2 |
| C18:2n6t | 1-100 | 1-1,000 | 2 |
| C18:3n6 | 5-100 | 5-1,000 | 10 |
| C18:3n3 | 5-100 | 5-5,000 | 10 |
| C20:0 | 0.25-100 | 0.25-1,000 | 1 |
| C20:1n9 | 5-100 | 5-1,000 | 10 |
| C20:2 | 1-100 | 1-1,000 | 2 |
| C20:3n6 | 5-100 | 5-1,000 | 10 |
| C20:3n3 | 0.25-100 | 0.25-1,000 | 0.5 |
| C20:4n6 | 10-100 | 10-1,000 | 20 |
| C20:5n3 | 5-100 | 5-1,000 | 10 |
| C21:0 | 5-100 | 5-1,000 | 10 |
| C22:0 | 0.25-100 | 0.25-1,000 | 1 |
| C22:1n9 | 0.25-100 | 0.25-1,000 | 0.5 |
| C22:2 | 1-100 | 1-1,000 | 2 |
| C22:6n3 | 1-100 | 1-1,000 | 2 |
| C23:0 | 0.25-100 | 0.25-1,000 | 1 |
| C24:0 | 5-100 | 5-1,000 | 10 |
| C24:1n9 | 1-100 | 1-1,000 | 2 |
