# Supplementary material for: Comprehensive and comparative lipidome analysis of Vitis vinifera L. cv. Pinot Noir and Japanese indigenous V. vinifera L. cv. Koshu grape berries
Source: PLoS One. 2017 Oct 20;12(10):e0186952. doi: 10.1371/journal.pone.0186952 (PMC5650187; doi:10.1371/journal.pone.0186952)
Supplement: S6 Table — (DOCX) [file pone.0186952.s013.docx]

| **S6 Table.** Recovered amounts, recovery rates, and coefficients of variation (CVs) for juice and skin samples supplemented with fatty acids. | | | |
| --- | --- | --- | --- |
|  | Recovered amount (µg/mL)* | Recovery rate (%)* | CV |
| Juice |  |  |  |
| C8:0 | 6.98 ± 0.13 | 83.0 ± 1.7 | 2.03 |
| C10:0 | 5.19 ± 0.06 | 96.9 ± 1.2 | 1.19 |
| C12:0 | 4.87 ± 0.08 | 92.8 ± 1.6 | 1.67 |
| C14:0 | 8.04 ± 0.46 | 114.3 ± 7.2 | 6.19 |
| C18:0 | 15.3 ± 0.17 | 98.8 ± 2.8 | 2.84 |
| C20:4n6 | 6.00 ± 0.37 | 107.0 ± 6.2 | 6.13 |
| Skin |  |  |  |
| C8:0 | 7.03 ± 0.40 | 84.5 ± 5.3 | 6.28 |
| C10:0 | 5.52 ± 0.30 | 103.2 ± 5.8 | 5.52 |
| C12:0 | 5.58 ± 0.47 | 102.6 ± 11.6 | 7.97 |
| C14:0 | 6.98 ± 0.10 | 103.3 ± 1.5 | 1.42 |
| C18:0 | 14.9 ± 0.23 | 114.7 ± 3.8 | 3.24 |
| C20:4n6 | 5.42 ± 0.18 | 96.7 ± 3.0 | 3.27 |
| * indicate means ± standard deviations calculated from three independent samples. | | | |
