# Supplementary material for: Comprehensive and comparative lipidome analysis of Vitis vinifera L. cv. Pinot Noir and Japanese indigenous V. vinifera L. cv. Koshu grape berries
Source: PLoS One. 2017 Oct 20;12(10):e0186952. doi: 10.1371/journal.pone.0186952 (PMC5650187; doi:10.1371/journal.pone.0186952)
Supplement: S7 Table — (DOCX) [file pone.0186952.s014.docx]

| **S7 Table.** Reproducibility and linearity of peak areas of main lipid components in Koshu skins. | | | | | | |
| --- | --- | --- | --- | --- | --- | --- |
| Compound | Skin volume (mg) | Peak area | | | | CV |
|  |  | Sample 1 | Sample 2 | Sample 3 | Average |  |
| LPC (16:0) | 5 | 3.15E+05 | 2.84E+05 | 3.05E+05 | 3.01E+05 | 5.3 |
|  | 10 | 6.03E+05 | 6.39E+05 | 6.19E+05 | 6.20E+05 | 2.9 |
|  | 15 | 9.11E+05 | 9.61E+05 | 8.86E+05 | 9.19E+05 | 4.2 |
|  | 20 | 1.24E+06 | 1.25E+06 | 1.21E+06 | 1.24E+06 | 1.6 |
| PC (34:2) | 5 | 4.47E+07 | 4.60E+07 | 4.85E+07 | 4.64E+07 | 4.2 |
|  | 10 | 8.82E+07 | 9.30E+07 | 8.75E+07 | 8.96E+07 | 3.3 |
|  | 15 | 1.13E+08 | 1.28E+08 | 1.21E+08 | 1.21E+08 | 6.2 |
|  | 20 | 1.65E+08 | 1.59E+08 | 1.47E+08 | 1.57E+08 | 5.9 |
| TG (54:7) | 5 | 2.22E+06 | 2.09E+06 | 2.32E+06 | 2.21E+06 | 5.3 |
|  | 10 | 5.47E+06 | 5.33E+06 | 5.63E+06 | 5.48E+06 | 2.8 |
|  | 15 | 8.29E+06 | 8.52E+06 | 8.50E+06 | 8.44E+06 | 1.5 |
|  | 20 | 1.19E+07 | 1.17E+07 | 1.15E+07 | 1.17E+07 | 1.9 |
